# NF-Y Recruits Both Transcription Activator and Repressor to Modulate Tissue- and Developmental Stage-Specific Expression of Human γ-Globin Gene

**DOI:** 10.1371/journal.pone.0047175

**Published:** 2012-10-10

**Authors:** Xingguo Zhu, Yongchao Wang, Wenhu Pi, Hui Liu, Amittha Wickrema, Dorothy Tuan

**Affiliations:** 1 Department of Biochemistry and Molecular Biology, Medical College of Georgia and College of Graduate Studies, Georgia Health Sciences University, Augusta, Georgia, United States of America; 2 Department of Medicine, University of Chicago, Chicago, Illinois, United States of America; Medical College of Wisconsin, United States of America

## Abstract

The human embryonic, fetal and adult β-like globin genes provide a paradigm for tissue- and developmental stage-specific gene regulation. The fetal γ-globin gene is expressed in fetal erythroid cells but is repressed in adult erythroid cells. The molecular mechanism underlying this transcriptional switch during erythroid development is not completely understood. Here, we used a combination of in vitro and in vivo assays to dissect the molecular assemblies of the active and the repressed proximal γ-globin promoter complexes in K562 human erythroleukemia cell line and primary human fetal and adult erythroid cells. We found that the proximal γ-globin promoter complex is assembled by a developmentally regulated, general transcription activator NF-Y bound strongly at the tandem CCAAT motifs near the TATA box. NF-Y recruits to neighboring DNA motifs the developmentally regulated, erythroid transcription activator GATA-2 and general repressor BCL11A, which in turn recruit erythroid repressor GATA-1 and general repressor COUP-TFII to form respectively the NF-Y/GATA-2 transcription activator hub and the BCL11A/COUP-TFII/GATA-1 transcription repressor hub. Both the activator and the repressor hubs are present in both the active and the repressed γ-globin promoter complexes in fetal and adult erythroid cells. Through changes in their levels and respective interactions with the co-activators and co-repressors during erythroid development, the activator and the repressor hubs modulate erythroid- and developmental stage-specific transcription of γ-globin gene.

## Introduction

The human β-like globin genes, consisting of embryonic ε-, fetal Gγ- and Aγ- and adult δ- and β-globin genes, are expressed in erythroid cells and undergo an ordered developmental switching program: ε-globin gene is expressed in early embryos until ∼7 weeks in gestation, when it is switched off and γ-globin genes are switched on; at the time of birth, γ-globin genes are switched off and β-globin gene is switched on. The mechanisms of transcriptional activation and silencing of human γ-globin genes have been under intensive investigation because of the clinical importance of γ-globin gene re-activation in the adult erythroid cells of sickle cell disease and β-thalassemia patients in ameliorating the symptoms of the diseases. Many pharmacological compounds have been found to re-activate fetal γ-globin gene in adult erythroid cells [1]. However, the mechanism by which these compounds re-activate γ-globin gene is not fully understood, as the molecular mechanism of γ-globin gene activation and subsequent inactivation during erythroid development has not been clearly established.

**Figure 1:**
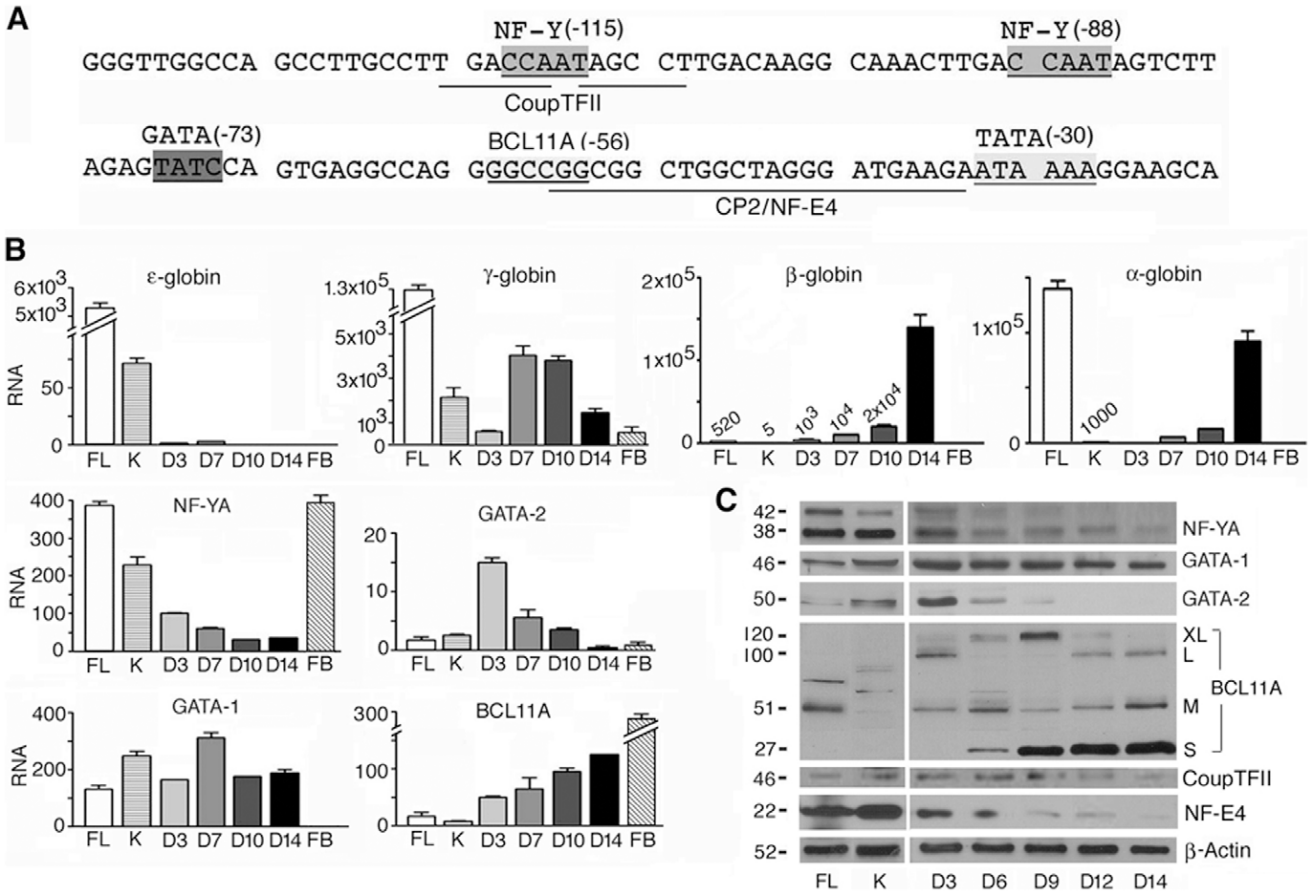
Expression profiles of human globin genes and transcription factors that bind to the proximal γ-globin promoter in human fetal and adult erythroid cells. **A.** Sequence of the proximal γ-globin promoter, which is identical in both GΓ- and Aγ-globin promoters. Shaded and/or underlined bases: DNA motifs that bind transcription factors as marked. Numbers in parentheses: first base positions in the motifs relative to the transcription start site. **B.** Transcription profiles of globin genes and transcription factors determined by quantitative real-time RT-PCR in total cellular RNAs isolated from human fetal liver, K562, and adult erythroid cells cultured from CD34+ cells for 3–14 days and non-erythroid human fetal brain cells, FL, K, D3–D14 and FB, respectively. In BCL11A panel, the PCR primer pair amplified all 4 different isoforms detected in Fig. 1C. The level of 18S ribosomal RNA set at 10^6^ served as the reference for comparison. RNA levels in K562, fetal liver and D14 cells were averages of two separate RNA preparations; RNA levels in other cells were averages of two RT-PCR reactions. **C.** Protein expression profiles of transcription factors determined by Western blots. Numbers in left margin: sizes of proteins in Kd.

Large-scale sequence analysis of human promoters finds that over 60% of annotated human promoters contain the CCAAT motifs, which are located frequently in the proximal promoter regions, 40–120 bases upstream of the TATA box [2], [3]. The CCAAT motifs are present in the proximal promoter regions of all globin genes (GenBank U01317, NG000006 and X14061). The CCAAT motif binds transcription factor NF-Y and plays a critical role in transcriptional activation and developmental switching of γ-globin genes [4]–[6]. NF-Y is a ubiquitously expressed protein complex composed of NF-YA, -YB and -YC subunits; YB and YC first associate through their histone fold domains to form a dimer, which then recruits YA–the regulatory subunit; the trimeric NF-Y through a DNA binding domain in YA binds to the CCAAT motif with specificity and affinity among the highest for DNA-binding transcription factors [3], [7], [8]. Among globin promoters, the proximal γ-globin promoter uniquely contains two tandem CCAAT motifs; in addition, it contains DNA motifs that bind erythroid transcriptional activators: GATA-2, as shown in this study, and the CP2/NF-E4 complex [9] and also general transcription repressors: BCL11A [10], [11] and COUP-TFII, an orphan nuclear receptor [12]–[15] (Fig. 1A). Base mutations in the DNA motifs in the proximal γ-globin promoter cause hereditary persistence of fetal hemoglobin (HPFH) [16]–[20], indicating the functional importance of these DNA motifs and the transcription factors recruited by them in regulating γ-globin gene expression. However, how these DNA-binding transcription factors interact to assemble the active γ-globin proximal promoter complex in fetal erythroid cells and the inactive promoter complex in adult erythroid cells is largely unknown.

In this study, we show that the developmentally-regulated, general activator NF-Y stably bound at the tandem CCAAT motifs served as an anchor to assemble a developmentally-regulated activator hub, NF-Y/GATA-2, which recruited co-activators CBP and MLL2 to modify histones [21], [22] and members of the Mediator complex [23] and basal transcription machinery to transcribe γ-globin mRNA and a repressor hub, BCL11A/COUP-TFII/GATA-1, which recruited co-repressor HDAC1 to antagonize the activities of the activator hub. The molecular assemblies of the active and inactive proximal γ-globin promoter complexes shed light on the molecular mechanism of γ-globin gene activation and repression during erythroid development and suggest potential mechanisms of pharmacological compounds in re-activating the repressed γ-globin gene in adult erythroid cells.

## Materials and Methods

### Human Cells and Ethics Statement

Normal human fetal livers ∼100 days in age were obtained from University of Washington Birth Defects Research Laboratory, which collects fetal tissues from aborted fetuses at regional hospital and clinics with protocols approved by the Human Subjects IRB of the University of Washington (Approval No. 11449) and provides the fetal tissues for research conducted by NIH-supported scientists. Human adult erythroid cells were grown from CD34+ cells isolated from peripheral blood samples of growth factor mobilized healthy donors as described, except without the FACS sorting step [24]. The human cells were obtained commercially from AllCells and processed with protocols approved by Human Assurance Committee of Georgia Health Sciences University (HAC file #10-09-064). K562 cells, obtained from ATCC, were cultured as described [25].

### Plasmids and Lentiviral Plasmids

For construction of plasmids and packaging of recombinant lentiviruses, see Methods S1.

### Transfection and Transduction

Transfection of GFP-reporter plasmids and transcription factor expression plasmids into K562 cells was carried out by electroporation. The level of GFP expression was determined after 48 hours from FACS dot-plots with correction for transfecion efficiencies as described [25], [26]. For transduction protocol of GFP-lentiviruses into primary erythroid cells, FACS analysis and sorting of the transduced GFP-fluorescent cells, see Methods S1.

### RNA Isolation, RT-PCR, Western Blots, Transfection and EMSA

RNA isolation, RT-PCR, Western blots, transfection and EMSA were carried out as described [25]–[27]. Human fetal brain RNA was from Biochain (Cat. # R124414).

### ChIP and Re-ChIP

ChIP and Re-ChIP were carried out as described [25], [28]. In ChIP, 10^6^ cells were used for each antibody pull-down and the pulled down chromatin was quantified by PCR.

### In vivo and in vitro co-IP, EMSA Probes, PCR Primers and Antibodies

See Methods S1.

## Results

### Expression Profiles of Human Globin Genes and Transcription Factors that Bind to the Proximal γ-globin Promoter in Primary Human Fetal and Adult Erythroid Cells

To study regulation of tissue- and developmental stage transcription of γ-globin gene, we investigated the proximal γ-globin promoter complex in human fetal liver erythroid cells, in which the γ-globin promoter is active, and adult erythroid cells cultured from CD34+ hematopoietic stem/progenitor cells for up to 14 days, during which, the progenitor cells undergo terminal differentiation to become mature, adult erythroid cells [24], in which the γ-globin gene is silenced. To molecularly characterize the cell systems, we determined the expression profiles in these primary cells of globin genes and transcription activators NF-Y, GATA-2 and CP2/NF-E4 and repressors COUP-TFII, GATA-1 and BCL11A that bind to the proximal γ-globin promoter (Fig. 1A).

The transcription profiles of globin mRNAs in fetal liver and adult erythroid cells demonstrated that these primary erythroid cells recapitulated the developmental globin gene switching program: In fetal liver erythroid cells, γ-globin genes were expressed at a high level but β-globin gene at a very low level, at ∼1% that of γ-globin genes; in contrast, in adult erythroid cells cultured for 14 days from CD34+ cells, γ-globin genes were suppressed and expressed at ∼1% that of the actively expressed β-globin gene (Fig. 1B, γ- and β-globin gene panels). In parallel with the decline of γ-globin gene expression during fetal to adult erythroid development, expression of transcription activators NF-YA, the regulatory and limiting subunit of NF-Y [3], GATA-2 and NF-E4 also declined (Fig. 1B and 1C). However, expression of repressors COUP-TFII, GATA-1 and BCL11A remained relatively stable or increased during fetal to adult erythroid development (Fig. 1C). The expression profile of BCL11A protein with differently-sized isoforms was complex: In fetal erythroid cells, only the median-sized isoforms were expressed at low levels; in Day 14 adult erythroid cells, the long-, median- and short-isoforms were all more abundantly expressed (Fig. 1B).

The human erythroleukemia K562 cells expressed γ- but not β-globin gene (Fig. 1B, top panels), like fetal erythroid cells, and the expression profiles of the transcription factors were also similar to those of fetal erythroid cells (Fig. 1B and 1C). Therefore, K562 cells were used in place of fetal erythroid cells for subsequent transfection experiments due to the availability of large numbers of easily transfectable cells.

### CCAAT and GATA Motifs Activate and GGCCGG Motif Represses γ-globin Promoter Activity in Erythroid Cells Regardless of their Developmental Stages

**Figure 2:**
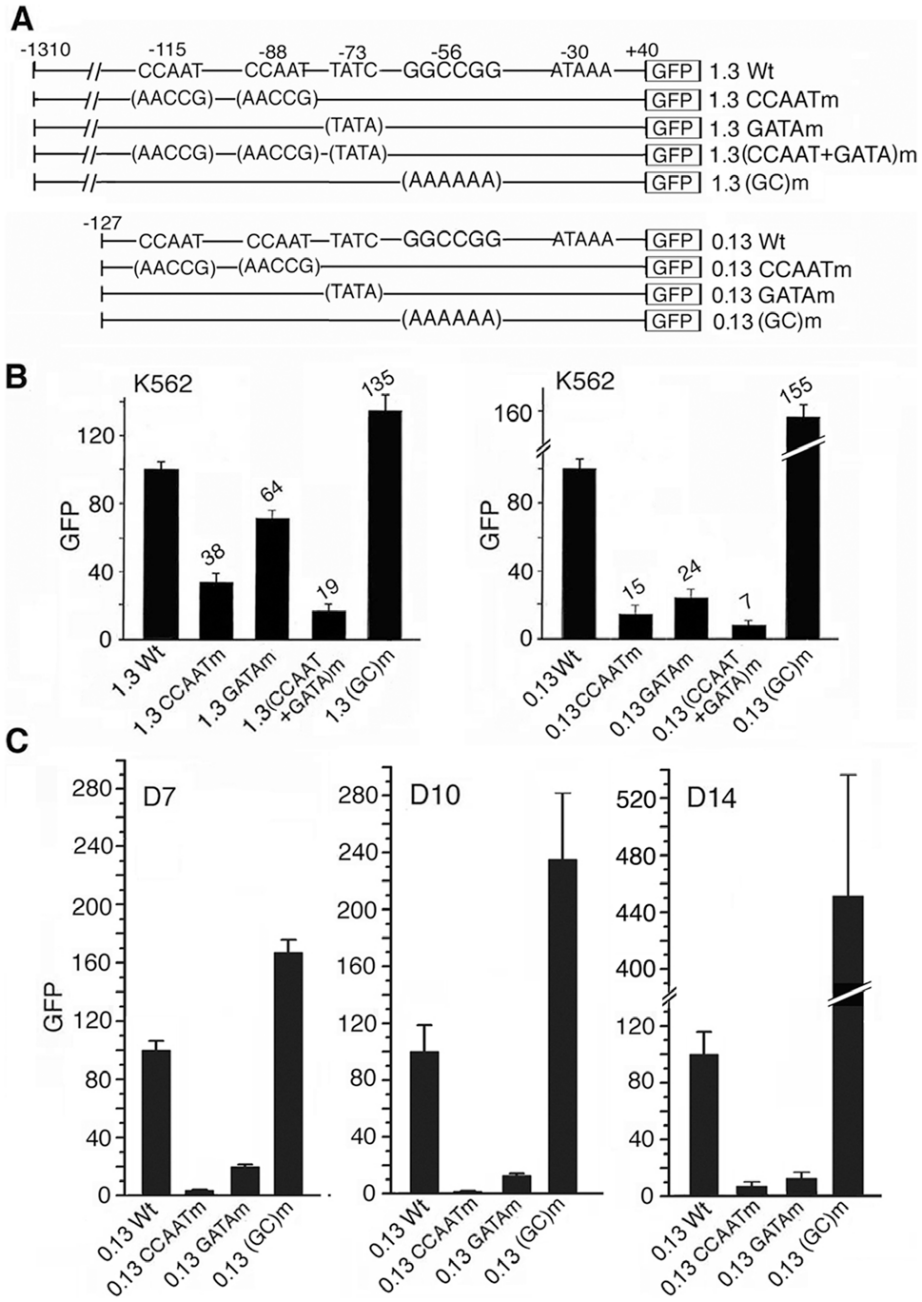
Function of the tandem CCAAT, GATA and GGCCGG motifs in regulating proximal γ-globin promoter activity, determined in GFP reporter plasmids by transfection/transduction assays. **A.** Plasmid maps of Wt and mutant long 1.3 kb and short, proximal 0.13 kb γ-globin promoter coupled to GFP gene. Mutated bases in the motifs were shown in parentheses. **B.** Transfection into K562 cells of GFP plasmids containing the long and the short promoters, left and right panels respectively. Numbers on top of the bars: fluorescence levels of GFP expressed from the mutant plasmids compared to those from Wt plasmids set at 100. Values were averages of two independent transcfection experiments. **C.** Transduction into adult erythroid cells of recombinant lentiviruses containing the Wt and mutant proximal γ-globin promoters coupled to GFP gene. Cells were transduced on day 3 and GFP fluorescence was determined on day 7, 10 and 14 of culture, as shown in the 3 panels respectively. The GFP level in control cells transduced with the wildtype 0.13 kb γ-globin promoter-GFP lentivirus was set at 100. Values were averages of two independent transduction experiments.

To functionally dissect the proximal γ-globin promoter, we focused on three DNA motifs near the TATA box: the tandem CCAAT motifs with flanking bases, which bind both activator NF-Y and repressor COUP-TFII, the −73 GATA site, whose functional significance was unknown until this study and the −56 GGCCGG motif, which binds repressor BCL11A [10], [11] (Fig. 1A). To determine the contribution of these motifs to promoter activity by transfection assays, we generated two sets of GFP reporter plasmids containing either the short 0.13 kb proximal promoter or the long 1.3 kb γ-globin promoter spanning the distal as well as the proximal promoter regions (Fig. 2A). In these wildtype (Wt) plasmids, mutations were introduced into the tandem CCAAT motifs to eliminate the binding site for NF-Y, which also eliminated the binding site for COUP-TFII, as the two binding sites overlapped at the CCAAT motif (Fig. 1A), or into the GATA or the GGCCGG motifs to eliminate binding of GATA factors or BCL11A (Fig. 2A). The plasmids and recombinant lentiviruses were transfected/transduced into K562 and primary adult erythroid cells, respectively.

The results showed that mutations of the CCAAT or the GATA motif significantly reduced the activities of the γ-globin promoter in both K562 and D7–14 primary adult erythroid cells (Fig. 2B and 2C). Thus, these two motifs bound transcription activators in both K562 and adult erythroid cells. Since the CCAAT mutation eliminated the binding of both activator NF-Y and repressor COUP-TFII, the drastically reduced activity of the mutant CCAAT promoter indicated that the CCAAT motif bound predominantly activator NF-Y over repressor COUP-TFII in both K562 and adult erythroid cells.

In contrast, base mutations in the GGCCGG motif drastically increased γ-globin promoter activity in both K562 and Day 7–14 adult erythroid cells (Fig. 2B and 2C), indicating that this motif was a repressive site in both K562 and adult erythroid cells. Since K562 cells, like fetal erythroid cells, expressed only BCL11A-M isoforms, these isoforms thus also bound to the GGCCGG motif and repressed γ-globin promoter activity. The results indicated that CCAAT and GATA motifs bound transcription activators and GGCCGG motif bound transcription repressors in erythroid cells regardless of their developmental stages.

### NF-Y Bound at the Tandem CCAAT Motifs Recruits and Stabilizes Binding of GATA-2 and BCL11A to the Neighboring GATA and GGCCGG Motifs, which Inherently cannot Bind or Bind Weakly the Respective Transcription Factors

**Figure 3:**
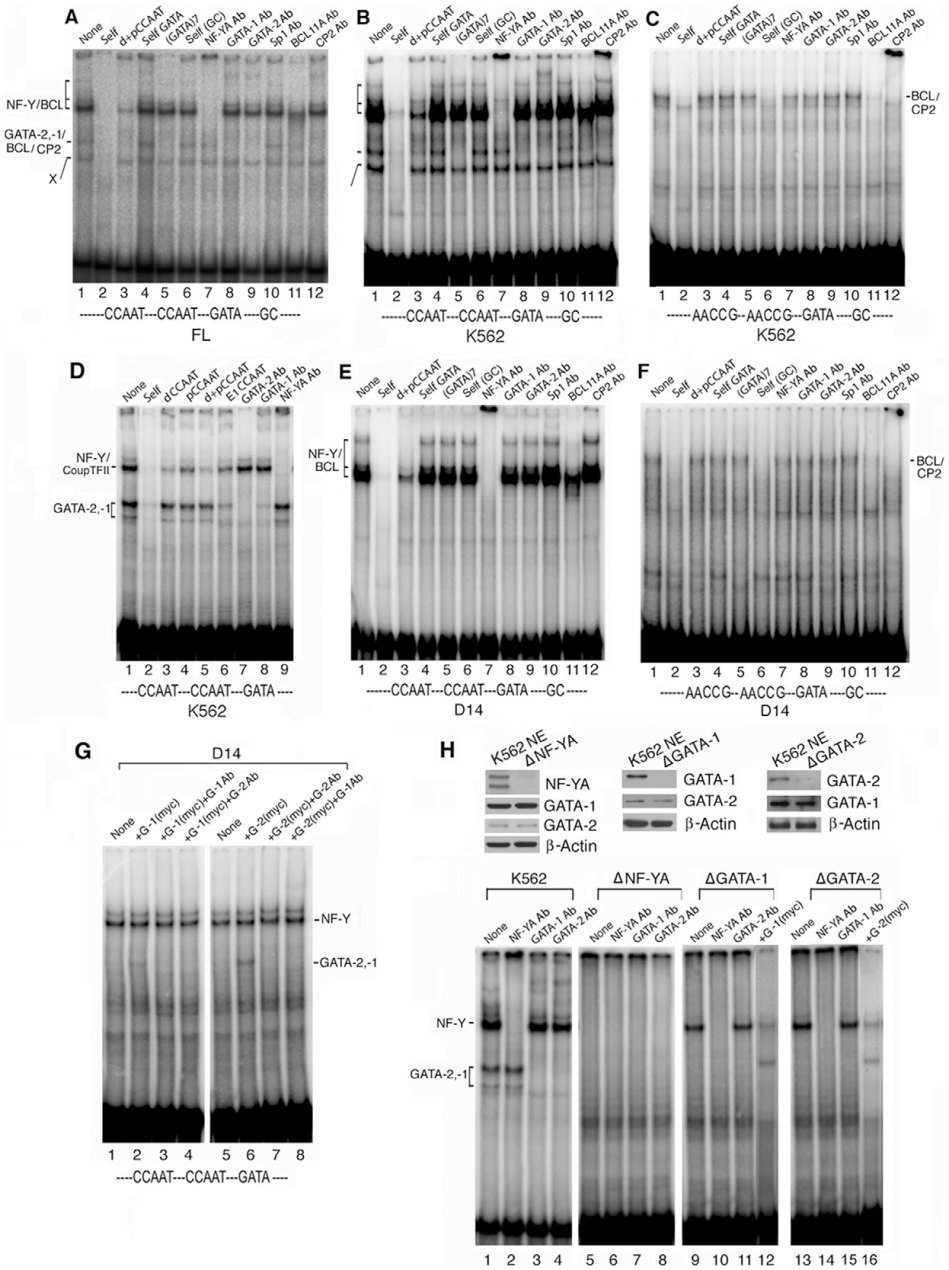
Identification of transcription factors that bind to the proximal γ-globin promoter as determined by EMSA. A, B & E. Wt proximal γ-globin promoter probe with nuclear extracts from human fetal liver, K562 cells and D14 adult erythroid cells, respectively. Top labels: Competitors and antibodies used in EMSAs. Self, self CCAAT, self GATA, (GATA)7 and self GC were respectively 100x molar excess of unlabeled probe, distal −115 and proximal −88 CCAAT motifs, the −73 GATA motif, seven tandem GATA motifs (29), and GC-rich bases spanning GGCCGG motif. Margin labels: bands generated by the transcription factors as marked. **C & F.** Mutant CCAAT proximal promoter probe with K562 and D14 nuclear extracts; all other labels were the same as in **A**. **D.** Binding affinity of COUP-TFII to its cognate site overlapping the distal CCAAT motif in the proximal γ-globin promoter: Competition EMSAs of proximal γ-globin promoter probe spanning the tandem CCAAT motifs to the GATA site (base positions: −120 to −62 in Fig. 1A) with K562 nuclear extract. Competitors d, p and d+p CCAAT: distal and proximal CCAAT motif and distal+proximal CCAAT motifs in the γ-globin promoter; E1CCAAT: CCAAT motif in the ERV-9 LTR enhancer upstream of the β-globin gene locus (25). For sequences of the competitors, see Methods S1. **G.** Binding affinities of GATA-1 and -2 to the proximal γ-globin promoter: EMSA of Wt proximal promoter probe with D14 nuclear extract. Lanes 2 & 6, 3 & 7, 4 & 8: myc-tagged GATA-1, G-1(myc), and myc-tagged GATA-2, G-2(myc), added respectively to D14 nuclear extract alone, and with GATA-1 or -2 antibody. **H.** Binding of GATA-1 and -2 to the proximal γ-globin promoter required presence of NF-Y determined by EMSA with immuno-depleted K562 nuclear extract (ID-EMSA). Upper panel: Western blots of K562 nuclear extracts immuno-depleted with antibody to NF-YA, GATA-1 and -2, ΔNF-YA, Δ GATA-1 and ΔGATA-2 respectively. Lower panel: EMSA of Wt proximal promoter probe spanning the tandem CCAAT motifs to the −73 GATA site with wildtype, ΔNF-YA, Δ GATA-1 and ΔGATA-2 K562 nuclear extracts respectively.

As it had not been shown whether NF-Y and BCL11A indeed bound to the CCAAT and GGCCGG motifs and which GATA factor(s) bound to the GATA motif in primary human erythroid cells, we carried out electrophoretic mobility shift assays (EMSAs) with nuclear extracts from human fetal liver, K562 cells and Day 14 adult human erythroid cells, using as probe the proximal γ-globin promoter of 94 bases from the CCAAT motifs to the CP2/NF-E4 site (Fig. 1A). With nuclear extracts from fetal liver and K562 cells, the probe generated three bands (Fig. 3 A and 3B, lanes 1). The top band was generated by NF-Y binding to the CCAAT motifs: the competitor spanning the tandem CCAAT motifs abolished most of the top band and NF-YA antibody completely super-shifted this band (Fig. 3A and 3B, lanes 3 and 7); however, antibodies to C/EBPγ or C/EBPδ, which could potentially bind to the CAAT motif, did not supershift the band (not shown). A small portion of this band contained BCL11A, since BCL11A antibody supershifted ∼30% of the NF-Y band (Fig. 3A and 3B, lanes 11; Fig. S1A). The GGCCGG motif serving as competitor, however, did not appreciably diminish the intensity of the NF-Y/BCL11A band (Fig. 3A and 3B, lanes 6), indicating that the GGCCGG motif by itself, even at 100x molar excess than the probe, was unable to bind significant amount of BCL11A to diminish the intensity of the NF-Y/BCL11A band. The GGCCGG motif did not bind Sp1, since Sp1 antibody did not supershift the NF-Y/BCL11A band (Fig. 3A&B, lane 10). Thus, NF-Y bound at the tandem CCAAT motifs appeared to recruit and stabilize binding of BCL11A to the GGCCGG site. On the other hand, the proximal γ-globin promoter with mutated GGCCGG motif bound much less BCL11A than did the Wt γ-promoter probe (Fig. S1B), indicating cooperation of the GGCCGG motif with the CCAAT motif in recruiting BCL11A to the proximal promoter. Since the NF-YA antibody completely super-shifted the NF-Y/BCL band (Fig. 3A and 3B, lanes 3 and 7), NF-Y and BCL11A appeared to co-exist in the same protein complex.

As NF-Y and COUP-TFII bound to the cognate sites that overlap at the distal CCAAT motif (Fig. 1A), the relative strengths of their binding were determined by competition EMSAs using as competitors the distal CCAAT motif (dCCAAT), which contained the COUP-TFII binding site, and the proximal CCAAT motif (pCCAAT), which did not contain the COUP-TFII site. The results showed that competitors dCCAAT and d+p CCAAT spanning both the COUP-TFII and NF-Y sites were 10% more efficient competitors than pCCAAT and E1CCAAT, spanning only the NF-Y site (Compare the NF-Y/COUP-TFII band intensities of lanes 3–6, Fig. 3D). Quantification of the NF-Y/COUP-TFII band intensities showed that the distal CCAAT motif was occupied predominantly by NF-Y, since COUP-TFII binding constituted only ∼10% of the NF-Y band (Fig. S1C).

The faster migrating band below the NF-Y band was generated by GATA-1 and -2, since the (GATA)7 competitor that binds GATA-1 and -2 [29] abolished this band, and both GATA-1 and -2 antibodies supershifted this band (Fig. 3A and 3B, lanes 5, 8 and 9), whereas GATA-3 antibody did not do so (not shown). Again, the oligonucleotide competitor spanning the −73 GATA site failed to diminish the intensity of the GATA band (Fig. 3A and 3B, lanes 4), and when used as a probe it did not bind the GATA factors to generate a GATA band (Fig. S1D). Thus, the −73 GATA site in the absence of the tandem CCAAT motifs was unable to bind the GATA factors. However, the proximal γ-globin promoter with mutated GATA motif bound much less GATA-2/−1 than did the Wt γ-promoter probe (Fig. S1D), indicating cooperation of the GATA motif with the CCAAT motif in recruiting GATA-2/−1 to the proximal promoter.

It was curious to note that although GATA-2 binding to the −73 GATA site required NF-Y bound at the neighboring CCAAT motif, suggesting that the GATA-2 EMSA band should also contain NF-Y bound at the neighboring CCAAT motif, yet the GATA-2 EMSA band did not appear to contain NF-Y, since the NF-Y antibody did not abolish or super-shift the GATA band (see Fig. 3A and 3B, lanes 7–9). One possible explanation for this apparent paradox was that the DNA/protein interaction detected in EMSA was not in a static state but in a dynamic equilibrium of protein/DNA and protein/protein interactions, as discussed previously [25]. In this network of dynamic association and dissociation reactions, NF-Y was first recruited to the CCAAT motif in the γ-globin promoter probe; the NF-Y protein/CCAAT DNA complex then recruited GATA-2 to the adjacent weak −73 GATA site to form a stable GATA-2 protein/GATA DNA complex; NF-Y was subsequently dissociated from the neighboring CCAAT site to bind to other un-occupied CCAAT sites in the free promoter probe, since the free probe was present in a large molecular excess over the transcription factor molecules in the EMSA reaction. Thus, the excess free probe was able to drive the dynamic EMSA reactions to an equilibrium, where the occupied probes contained predominantly only one bound protein per probe to generate either the GATA-2/−1 or the NF-Y band observed in the EMSA gels.

In D14 adult erythroid cells, the proximal promoter probe generated only an NF-Y band, since the tandem CCAAT motifs as competitors abolished the band and NF-Y antibody completely super-shifted the band (Fig. 3E, lanes 3 and 7). Approximately 40% of the NF-Y band contained BCL11A, since BCL11A antibody supershifted ∼40% of the band (Fig. 3E, lanes 7 and 11; Fig. S1A). Even though D14 adult erythroid cells expressed abundant GATA-1 protein (Fig. 1C), GATA-1 was not recruited by NF-Y to the −73 GATA site to generate a GATA band (Fig. 3E, lanes I, 5, 8 and 9), indicating that in the absence of a detectable level of GATA-2 in D14 cells (Fig. 1C), GATA-1 was not recruited to the −73 GATA site. Thus, GATA-1 recruitment appeared to require GATA-2 bound at the −73 GATA site. Indeed, addition to D14 nuclear extract of myc-tagged GATA-2, but not myc-tagged GATA-1, generated a clear GATA band (Fig. 3G, lanes 2 and 6). As this GATA band was completely abolished by either GATA-2 or -1 antibody (Fig. 3G, lanes 3&4 and 7&8), GATA-2 and -1 appeared to form a hetero-dimer at the −73 GATA site.

With both K562 and D14 nuclear extracts, the mutant proximal promoter probe containing mutated CCAAT motifs generated no NF-Y band as anticipated, but also no GATA band and only two faint bands that were super-shifted by BCL11A and CP2 antibodies (Fig. 3C and 3F, lanes 3, 7–11; Fig. S1A). This again demonstrated that in the absence of NF-Y bound at the CCAAT motifs, the mutant promoter containing normal GATA and GGCCGG motifs was unable to efficiently bind GATA-2/−1 and BCL11A in both K562 and D14 erythorid cells.

To further investigate the role of NF-Y in recruiting GATA-2 and/or -1, we carried out ID-EMSA with K562 nuclear extract from which NF-Y, GATA-2 or -1 was immuno-depleted (Fig. 3H, top). In ID-EMSA with the NF-YA depleted (ΔNF-YA) nuclear extract, the proximal γ-globin promoter probe generated neither the NF-Y band nor the GATA bands (Fig. 3H, lane 5), although GATA-2 and -1 proteins were present in the ΔNF-YA extract (Fig. 3H, top left panel). This result again confirmed that recruitment of GATA-2/−1 to the −73 GATA site required NF-Y bound at the neighboring CCAAT motifs. In contrast, the ΔGATA-1 and -2 nuclear extracts generated the NF-Y band with the γ-globin promoter probe (Fig. 3H, lanes 9 and 13), indicating that NF-Y binding to the promoter probe did not require presence of either GATA-2 or -1. Addition of myc-tagged GATA-1 or -2 to these nuclear extracts regenerated the GATA band (Fig. 3G, lanes 12 and 16).

Together, the EMSA results indicated a hierarchical binding order of the transcription factors to the proximal promoter: NF-Y bound strongly at the CCAAT motifs formed a stable anchor that subsequently recruited GATA-2 and BCL11A to their cognate sites; the bound GATA-2 in turn recruited GATA-1. The results also indicated that the proximal γ-globin promoter bound both transcription activator NF-Y and repressor BCL11A in both fetal liver and adult erythroid cells, regardless of whether the γ-globin promoter was active or repressed in the respective erythroid cells.

### NF-Y and GATA-2 Activate and GATA-1 and BCL11A Repress γ-globin Promoter Activity

**Figure 4:**
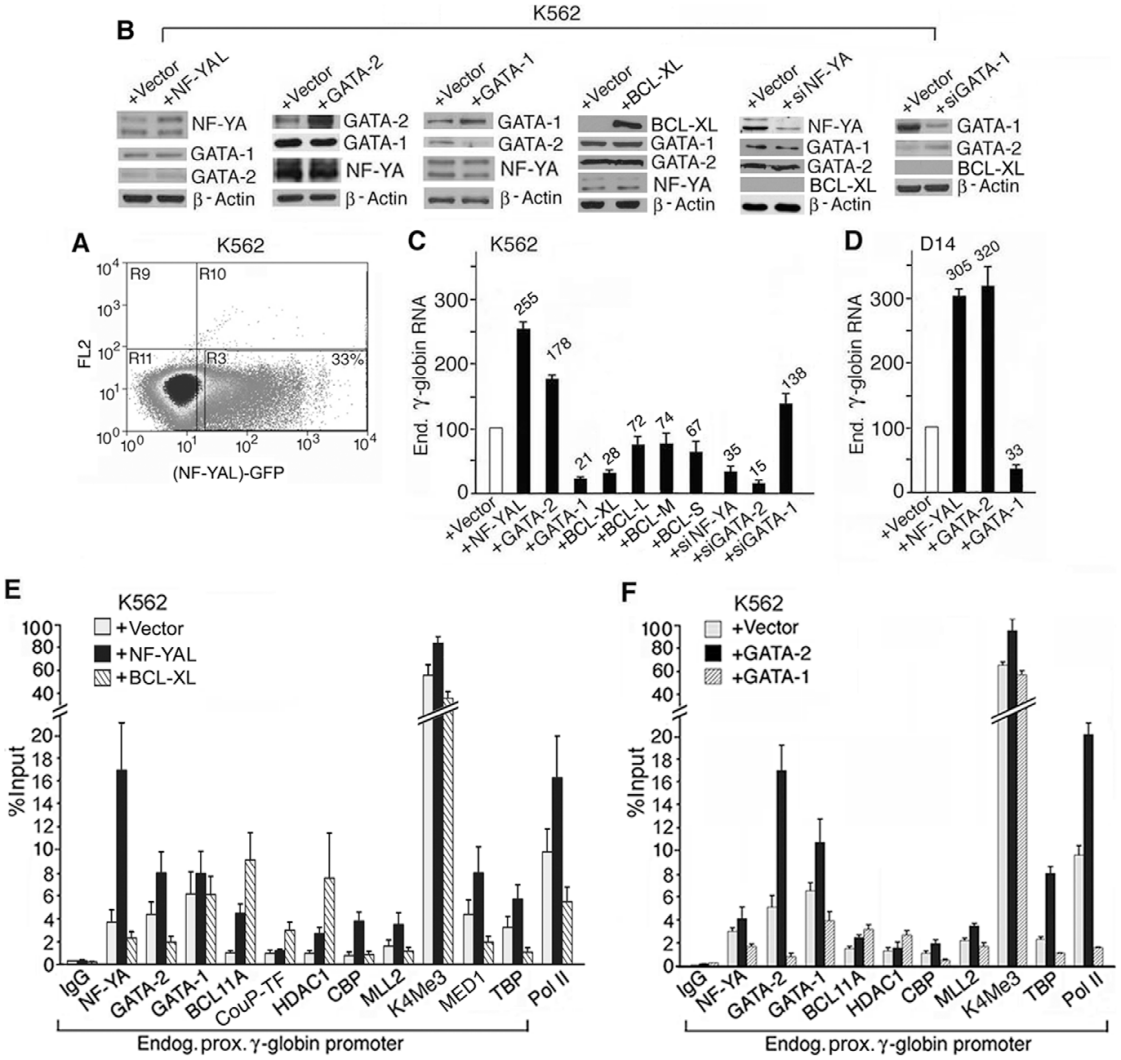
Over-expression of NF-YA, GATA-2, -1 or BCL11A activates or represses γ-globin promoter activity and induces corresponding changes in the *in vivo* assembly of proximal γ-globin promoter complex. **A.** Fluorescent K562 cells over-expressing NF-YA long isoform (YAL) with co-expressed GFP sorted by FACS. Lower right quadrant: sorted GFP-fluorescent cells comprising 33% of total cell population used for analyses in panels **B–E.** FACS sorting of K562 and D14 cells over-expressing the other three transcription factors (not shown). B. Western blots of proteins isolated from K562 cells over-expressing NF-YAL, GATA-2, -1 or BCL-XL and K562 cells in which NF-YA or GATA-1 was knocked down by the respective siRNA, siNF-YA or siGATA-1. +Vector: control K562 cells trasduced or transfected by the empty vector. **C & D.** Effects of over-expression/knockdown of NF-YAL, GATA-2, -1 or various isoforms of BCL11A on the transcription level of endogenous γ-globin mRNA in K562 and D14 adult erythroid cells determined by real-time RT-PCR. The level of γ-globin mRNA in K562 and D14 cells transduced/transfected with the vector was set at 100. Levels of γ-globin mRNA in test samples were averages of two independently transduced/transfected cells. Effects of over-expressing NF-YA short isoform, NF-YAS, on transcription of γ-globin mRNA in K562 and D14 cells were similar to the effects of over-expressing NF-YAL (not shown). **E & F**. ChIP assays of the endogenous γ-globin promoter in K562 cells over-expressing NF-YAL, BCL-XL, GATA-2 or -1. The NF-YA and BCL11A antibodies used in the ChIP assays recognized all isoforms of the respective transcription factor (Fig. 1C) and should pull down chromatin associated with all the isoforms of NF-YA and BCL11A. ChIP values were averages of two independent pull-down assays.

To determine the functional roles of NF-Y, GATA-2 and -1 and BCL11A on γ-globin promoter activity, we over-expressed or knocked-down by siRNA the respective transcription factors and measured the resultant effects on transcription of the endogenous γ-globin mRNA in K562 and in primary adult erythroid cells. The transcription factors were either over-expressed or siRNA knocked down with a co-expressed GFP selectable marker gene in a lentiviral vector for NF-YA, GATA-1 and -2 or as a GFP fusion gene in a plasmid vector for BCL11A. The transfected K562 cells and the transduced Day 14 adult erythroid cells were sorted by FACS (Fig. 4A). The sorted fluorescent cells were then used for Western blot to assess the degree of over-expression or knockdown of the respective transcription factors (Fig. 4B) and qRT-PCR to determine the subsequent effects on transcription of the endogenous γ-globin mRNA. The protein and RNA analyses showed that over-expression of NF-YA, either the long NF-YAL or the short NF-YAS isoform (see Fig. 1C), and of GATA-2 activated while over-expression of GATA-1 suppressed transcription of γ-globin mRNA in both K562 and D14 adult erythroid cells (Fig. 4C and 4D; NF-YAS results not shown). Thus, NF-Y and GATA-2 were activators while GATA-1 was a repressor of γ-globin promoter activity in both K562 and D14 cells independent of the developmental stages of the cells. In addition, over-expression of each of the four BCL11A isoforms–XL, L, M and S–in K562 cells all suppressed transcription of γ-globin mRNA, with the XL isoform being the most effective repressor (Fig. 4C), in agreement with earlier findings [10], [30]. Consistent with the over-expression results, knockdown of NF-Y and GATA-2 conversely suppressed whereas knockdown of GATA-1 conversely activated transcription of γ-globin mRNA (Fig. 4C). Thus, NF-Y and GATA-2 were activators and GATA-1 and BCL11A were repressors of γ-globin promoter activity.

### Over-expression of Transcription Activator NF-Y or GATA-2 or Repressor BCL11A or GATA-1 Changes the *in vivo* Assembly of the Proximal γ-globin Promoter Complex

To determine whether changes in transcription of γ-globin mRNA induced by the over-expressed transcription activators or repressors were correlated with corresponding changes in the *in vivo* assembly of the proximal γ-globin promoter complex, we carried out ChIP assays on the sorted fluorescent cells over-expressing each of the transcription factors. ChIP results showed that over-expression of NF-YA, the regulatory and limiting subunit of NF-Y [3], increased occupancy of NF-Y on the proximal γ-globin promoter (Fig. 4E). As a result, GATA-2 occupancy also increased (Fig. 4E), due likely to increased recruitment by NF-Y of GATA-2 to the −73 GATA site, even though the level of GATA-2 was not increased by over-expression of NF-Y (Fig. 4B, first panel). Associated with the increased occupancies of activators NF-Y and GATA-2, occupancies increased also for the following co-activators (Fig. 4E): CBP with histone acetyltransferase activity [21] that can bind to GATA-2 [31], MLL2 with histone methyltransferase activity [22], which could be recruited to the globin gene locus by the transcription activators [32] to methylate lysine 4 in histone 3 and generate the H3K4me3 chromatin mark associated with actively transcribed gene locus, and Mediator 1, a member of the Mediator complex that has been shown to interact with GATA-2 in mouse erythroid and thyroid cells [33], [34] and may mediate signal transmission between the activators and the basal transcription machinery. Occupancies of TBP and Pol II that could be recruited by NF-Y, GATA-2 and Mediator 1 to the TATA box [3], [35] also increased at the proximal γ-globin promoter (Fig. 4E). Thus, over-expression of NF-Y increased occupancy of NF-Y, which in turn increased occupancies of GATA-2, the co-activators and TBP and Pol II, resulting in increased transcription of γ-globin mRNA (Fig. 4C).

To determine whether co-activators MLL2 and Mediator 1 indeed regulated transcription of γ-globin gene, the expression of MLL2 or Mediator 1 was knocked down by the respective siRNAs in K562 cells. Transcription of γ-globin mRNA was reduced by 60–80% as a result of the knockdown of either MLL2 or Mediator 1 (Fig. S2), indicating the critical importance of MLL2 and MED 1 in transcriptional regulation of γ-globin promoter activity in human erythroid cells.

Over-expression of NF-YA increased occupancies also of repressor BCL11A and co-repressor HDAC1 at the proximal promoter (Fig. 4E), due likely to recruitment of BCL11A by the increased occupancy of NF-Y, since NF-Y bound at the tandem CCAAT motifs was able to interact with and recruit BCL11A to the neighboring GGCCGG motif, as shown by EMSA (Fig. 3A, 3B and 3E); increased occupancy of BCL11A in turn recruited more HDAC1 due likely to BCL11A/HDAC1 interaction [30]. Since BCL11A-M, the major isoform detectable in K562 cells (Fig. 1C), was a weak transcriptional repressor (Fig. 4C), presence of the weak repressor complex BCL11A-M/HDAC1 in the proximal promoter complex did not overcome the activity of the induced activator complex NF-Y/GATA-2, which activated transcription of γ-globin mRNA at a higher level as a result of NF-Y over-expression (Fig. 4C). Consistent with these findings, knockdown of NF-YA in K562 cells decreased occupancy of not only NF-Y but also GATA-2; however, NF-Y knockdown caused not a decrease in occupancy of BCL11A as anticipated from NF-Y/BCL11A interaction but a slight increase in BCL11A occupancy (Fig. S3A). This was likely due to strong interaction of BCL11A with COUP-TFII [11], whose occupancy at the proximal γ-globin promoter increased as a result of NF-Y knockdown (Fig. S3A). Since COUP-TFII binding site overlapped with the NF-Y binding site in the proximal γ-globin promoter (Fig. 1A), COUP-TFII was able to bind competitively to the proximal promoter at a higher level as a result of the decrease in occupancy of NF-Y. Thus, NF-Y knockdown decreased occupancies of activators NF-Y and GATA-2 and co-activator MLL2 but increased occupancies of repressors COUP-TFII and BCL11A and co-repressor HDAC1 at the proximal promoter (Fig. S3A), leading to transcriptional suppression of γ-globin mRNA (Fig. 4C).

On the other hand, over-expression of strong repressor BCL11A-XL in K562 cells not only increased occupancies of repressors BCL11A and COUP-TFII and co-repressor HDAC1 but also reduced occupancies of activators NF-Y and GATA-2 and co-activators CBP, MLL2 and MED1 and TBP and Pol II (Fig. 4E). The combination of the strong repressor complex BCL-XL/COUP-TFII/HDAC1 with the weakened activators/co-activators complex thus drastically reduced transcription of γ-globin mRNA due to BCL-XL over-expression (Fig. 4C).

Over-expression of activator GATA-2 and repressor GATA-1 induced respective changes in occupancies of the transcription activators, repressors, co-activators and co-repressors (Fig. 4F) similar to the respective changes induced by over-expression of NF-Y and BCL11A (Fig. 4E). Notably, over-expression of GATA-2 increased occupancy not only of GATA-2 but also of GATA-1 on the proximal γ-globin promoter (Fig. 4F), even though GATA-1 expression remained the same in cells over-expressing GATA-2 as in control cells (Fig. 4B, 2^nd^ panel from left). Thus, over-expression of GATA-2 resulted in higher occupancy of not only GATA-2 but also GATA-1. Paradoxically, over-expression of GATA-1 decreased occupancy of GATA-1 as well as of GATA-2 (Fig. 4F). Since GATA-2 expression was suppressed by GATA-1 over-expression (Fig. 4B, 3^rd^ panel from left), the decreased cellular level of GATA-2 reduced occupancy of GATA-2, which led in turn to decreased recruitment of GATA-1, despite GATA-1 over-expression. Thus, GATA-1 recruitment to the proximal γ-globin promoter depended on GATA-2 occupancy, in agreement with earlier EMSA results (Fig. 3E and 3G).

It could be argued that the changes in chromatin occupancies due to over-expression of GATA-2 and -1 might not have occurred at the proximal γ-globin promoter as was interpreted above but occurred at the multiple GATA sites in the distal promoter immediately upstream of the proximal promoter, since the in vivo ChIP assays might not be able to differentiate between the distal and proximal GATA sites separated by short distances of ∼100 DNA bases. To address this question, we carried out ChIP assays of transfected plasmid containing no distal promoter and only the 0.13 kb proximal γ-globin promoter linked to the GFP reporter gene. The results showed that over-expression of GATA-1 or -2 caused similar changes in the occupancies of transcription factors and co-factors on the transfected 0.13 kb proximal promoter as on the endogenous γ-globin proximal promoter (compare Fig. S3B and Fig. 4F). These results supported the original interpretation that changes in the levels of transcription factors caused changes in the assembly of the proximal γ-globin promoter complex.

In summary, the ChIP assays showed that changes in the levels of transcription activators or repressors induced distinct changes in the proximal γ-globin promoter complex in correlation with the induced activation or repression of γ-globin promoter activity: Over-expression of activators induced higher occupancies of the activators and co-activators but also of the repressor and co-repressor, which were apparently weak and did not prevent the activators/co-activators from activating transcription of γ-globin mRNA. On the other hand, over-expression of repressors not only increased occupancies of the repressors and co-repressor but also reduced occupancies of activators and co-activators; thus, the combination of a strong repressor complex with a weakened activator complex repressed transcription of γ-globin mRNA. Both activators and repressors were present in both the activated and the repressed γ-globin promoter complex, due at least in part to interaction between the activator and the repressor: NF-Y with BCL11A and GATA-2 with GATA-1, as was indicated also by EMSA results (Fig. 3A, 3B and 3E). Thus, γ-globin promoter activity appeared to be determined by the relative abundance/strengths of the activator/co-activator complex vs. the repressor/co-repressor complex.

### Molecular Assemblies of the Active and the Repressed Proximal γ-globin Promoter Complexes in Human Fetal and Adult Erythroid Cells

**Figure 5:**
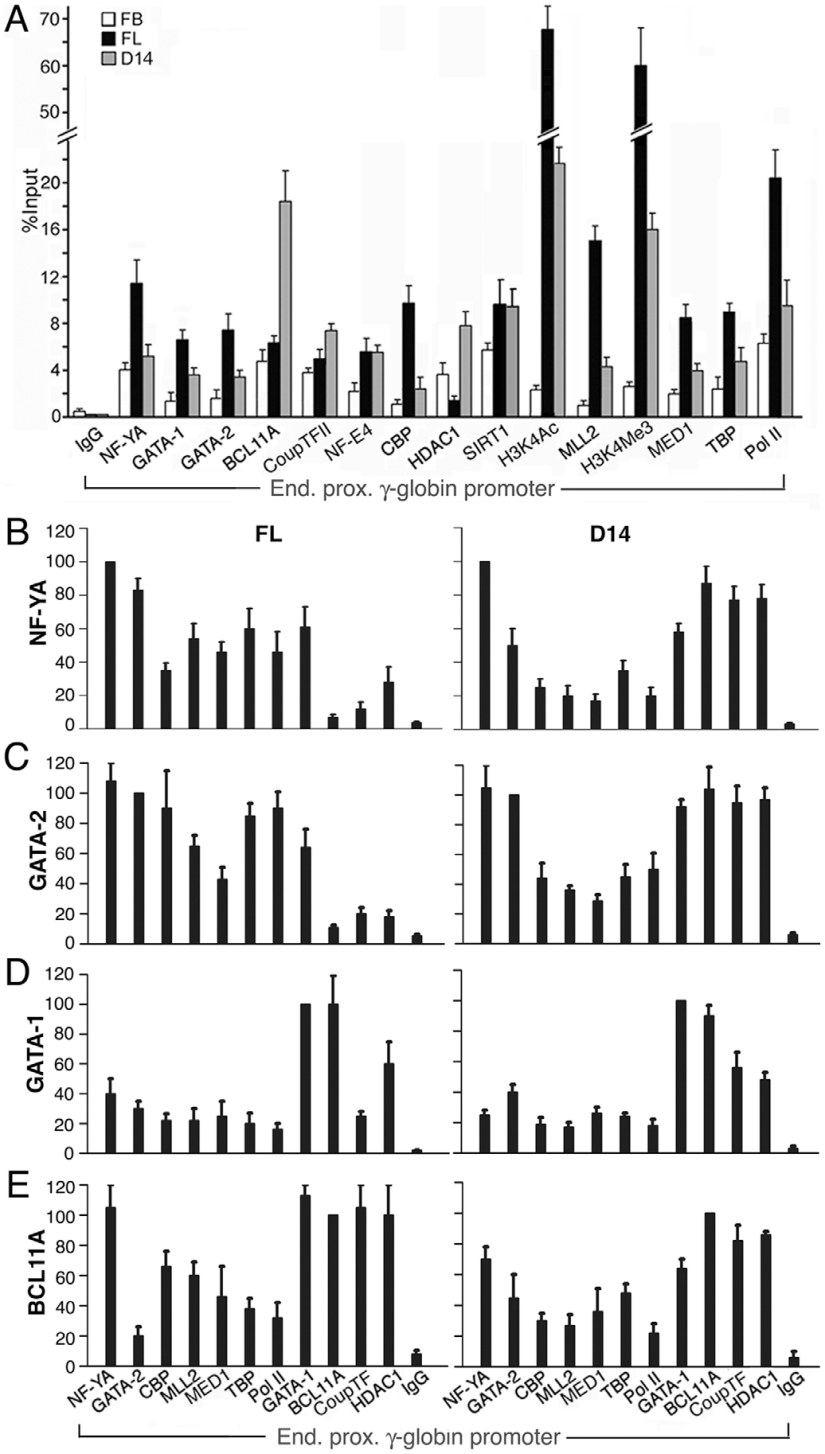
Molecular assemblies of the active and repressed proximal γ-globin promoter complexes in human fetal and adult erythroid cells. **A.** ChIP assays of the proximal γ-globin promoter complexes in fetal brain, fetal liver and adult D14 erythroid cells, FB, FL and D14, respectively. Values were averages of two independent pull-down assays. **B–E.** Re-ChIP assays of fetal liver and D14 adult erythroid cells, left and right panels respectively: The antibodies to the respective transcription factors used in first ChIP is shown on the Y-axis. The amount of γ-globin promoter pulled down by antibodies in the 1st ChIP was set at 100 to serve as the reference for comparing the amount of promoter pulled down in the 2nd ChIP by antibodies to proteins shown on the X-axis. Values were averages of two independent re-ChIP assays.

To verify the molecular assembly of the activated and repressed proximal γ-globin promoter complexes obtained in K562 cells due to over-expression of transcription activators NF-Y and GATA-2 or repressors BCL11A and GATA-1 (Fig. 4), we examined by ChIP the *in vivo* assembly of the active and the repressed proximal γ-globin promoter complexes in primary human fetal and adult erythroid cells, in which the transcription activators and repressors were developmentally regulated (see Fig. 1B and 1C). In fetal liver erythroid cells, as compared to human fetal brain cells in which the globin gene locus is transcriptionally inactive, the active, proximal γ-promoter bound activators NF-Y, GATA-2 and NF-E4 and also repressors BCL11A and COUP-TFII (Fig. 5A). The activators recruited co-activators CBP and MLL2 to generate high levels of active chromatin marks H3K4Ac and H3K4Me3, and Mediator 1, TBP and Pol II (Fig. 5A) to actively transcribe γ-globin gene. However, the repressors BCL11A and COUP TFII appeared unable to recruit a significant level of HDAC1 in fetal erythroid cells (Fig. 5A).

In contrast, in D14 adult erythroid cells, which expressed lower levels of activators NF-Y and GATA-2 but higher levels of BCL11A and GATA-1 as compared to fetal erythroid cells (Fig. 1B and 1C), the repressed γ-globin promoter bound much lower levels of NF-Y and GATA-2 and -1 and co-activators CBP and MLL2 and TBP and Pol II but much higher levels of BCL11A and HDAC1 (Fig. 5A). Even though GATA-1 was expressed at a higher level in adult D14 erythroid cells than in fetal erythroid cells, GATA-1 occupancy was lower in D14 cells than in fetal erythroid cells (Fig. 5A), due again to dependence of GATA-1 recruitment on GATA-2 occupancy, which was low since GATA-2 protein was expressed at an undetectable level in adult D14 erythroid cells (Fig. 1C). It was curious that even though GATA-2 expression was undetectable in D14 erythroid cells by Western blot, GATA-2 was detected by in vivo ChIP assay to be present in the repressed proximal γ-globin promoter complex, in opposition to the findings of the in vitro EMSA that the proximal γ-globin promoter probe did not bind GATA-2 or -1 in the D14 nuclear extract (Fig. 3E). An explanation for the different *in vivo* and *in vitro* results could be that even though GATA-2 was expressed at a very low level in adult D14 erythroid cells, the expressed GATA-2 protein existed *in vivo* in the nucleus in discreet nuclear structures [36] with relatively high local concentrations of GATA-2 that were detectable by the ChIP assays. However, when the nuclear structure was disrupted to make the protein/nuclear extracts for Western blot and EMSA, the homogenized GATA-2 concentration in the bulk extracts could be too low to be detectable by either of the two *in vitro* methods. In both fetal and adult erythroid cells, ChIP assays showed that the levels of NF-E4 and SIRT1–an HDAC that associates with BCL11A [37]–did not change significantly in the proximal promoter complexes (Fig. 5A). These proteins appeared not to be involved in the developmental repression of the γ-globin promoter in D14 adult erythroid cells and were not further investigated.

In summary, ChIP assays in primary fetal and adult erythroid cells demonstrated that the molecular assemblies of the proximal promoter complexes, as measured by the relative occupancies, therefore abundance/strengths, of the activators/co-activators vs. the repressors/co-repressor, correlated with the developmental expression profiles of the transcription factors and with γ-globin promoter activities in fetal and adult erythroid cells.

To examine the *in vivo* interactions of NF-YA, GATA-2, and -1 and BCL11A among one another and with other proteins in the active and the repressed γ-globin proximal promoter complexes, we next carried out re-ChIP assays of the chromatin initially pulled down by the antibody to each of these four factors from fetal and adult erythroid cells in the first ChIP. In fetal erythroid cells, the re-ChIP results showed that activators NF-YA and GATA-2 associated with each other and with the co-activators at higher levels than with repressors BCL11A and Coup-TFII and co-repressor HDAC1 (Fig. 5B & 5C, left panels), whereas the repressors BCL11A and Coup-TFII associated with each other and with co-repressor HDAC1 at higher levels than with the activators and the co-activators (Fig. 5D & 5E, left panels), except for BCL11A, which also associated at a high level with NF-YA (Fig. 5E, left panel, BCL11A and NF-YA lanes), and for GATA-1, which associated at high levels with both activators NF-YA and GATA-2 and repressor BCL11A (Fig. 5B–E, left panels, GATA-1 lanes).

In re-ChIP of the repressed γ-globin promoter complex in Day 14 adult erythroid cells, the activators appeared to associate at lower levels with the co-activators but at higher levels with the repressors and the co-repressor (Fig. 5B & 5C, right panels), whereas the repressors BCL11A and CoupTFII still associated with each other and with the co-repressor at higher levels than with the activators and the co-activators (Fig. 5D and 5E, right panels), again except for BCL11A, which associated at a high level with NF-YA (Fig. 5E, right panel, BCL11A and NF-YA lanes), and for GATA-1, which associated at high levels with both activators NF-YA and GATA-2 and repressor BCL11A (Fig. 5B–E, right panels, GATA-1 lanes).

In summary, ChIP assays showed that in the active γ-globin promoter complex of fetal erythroid cells, occupancies of transcription activators NF-YA, GATA-2 and co-activators CBP, MLL2, Med 1, TBP and pol II were higher than those of transcription repressors BCL11A and CoupTFII and co-repressor HDAC1; however, in the repressed γ-globin promoter complex of D14 adult erythroid cells, occupancies of transcription activators and co-activators were lower than those of transcription repressors and co-repressor (Fig. 5A). Re-ChIP assays confirmed that in the active promoter complex, the activators associated at higher levels with co-activators than with repressors and co-repressors (Fig. 5B & 5C, left panels); in the repressed γ-globin promoter complex, the activators associated at higher levels with the repressors and co-repressor than with the co-activators (Fig. 5B & 5C, right panels). In contrast, the repressors and co-repressor associated preferentially with each other in both the active and the repressed promoter complexes (Fig. 5D and 5E, right and left panels). Through cross interactions of the activators with the repressors, NF-Y with BCL11A and GATA-2 with GATA-1, both the activators/co-activators and the repressors/co-repressor were present in both the active and the repressed γ-globin promoter complexes in fetal and adult erythroid cells (Fig. 5A and 5B).

### Protein Interaction Network in the Assembly of the Activator and the Repressor Hubs

**Figure 6:**
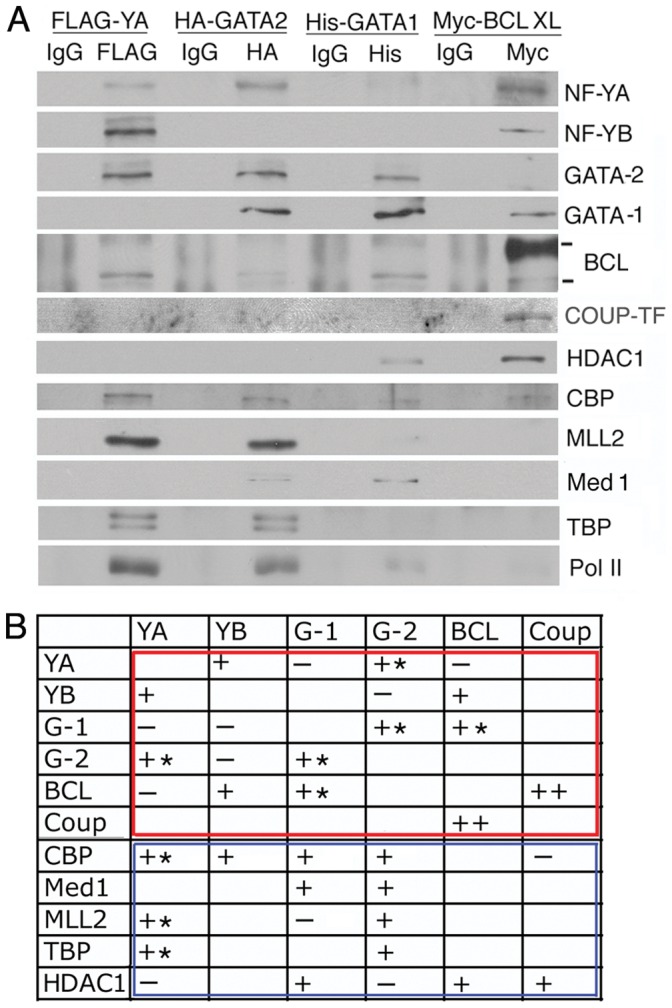
Protein interaction network in the proximal γ-globin promoter complex. A. Western blots of proteins co-immunoprecipitated with tagged NF-YA, GATA-2 or -1 or BCL11A-XL expressed from plasmids transfected into K562 cells. **B.** Pair-wise in vitro interactions between purified transcription factors and co-factors. Pair-wise interactions within the red frame: in vitro interactions between transcription factors. Pair-wise interactions within the blue frame: in vitro interactions between transcription factors and co-factors. ++, +, −: strong, average or no interaction between the proteins. Interactions marked with ∗: the proteins in the pair-wise interaction were isolated from both K562 and HEK293 transfected with the expression plasmids for the target proteins. Otherwise, the proteins were isolated from transfected HEK293 cells.

Since ChIP and re-ChIP assays showed association of the transcription factors and co-factors with γ-globin promoter DNA but did not reveal whether the component proteins interacted with one another, we next carried out *in vivo* and *in vitro* co-immunoprecipitation (co-IP) to investigate the protein interaction network in the proximal promoter complex. For *in vivo* co-IP, the four key transcription factors, NF-YA, GATA-2 and -1 and BCL-XL were expressed with different tags from plasmids separately transfected into K562 cells. The proteins associated with each of the tagged transcription factors were pulled down by the specific antibodies for each of the tags. Western blots of pulled-down proteins showed particular protein association patterns: Activators NF-YA and GATA-2 associated with each other and with co-activators CBP and MLL2 and TBP and pol II but not with co-repressor HDAC1; in addition, activators NF-YA and GATA-2 cross-associated with repressors BCL11A and GATA-1 respectively (Fig. 6A, 1^st^ and 2^nd^ columns from left). In contrast, repressor BCL-XL associated only with co-repressor HDAC1 but not with the co-activators, and repressor COUP-TFII associated only with BCL11A (Fig. 6A, 4^th^ column).

**Figure 7:**
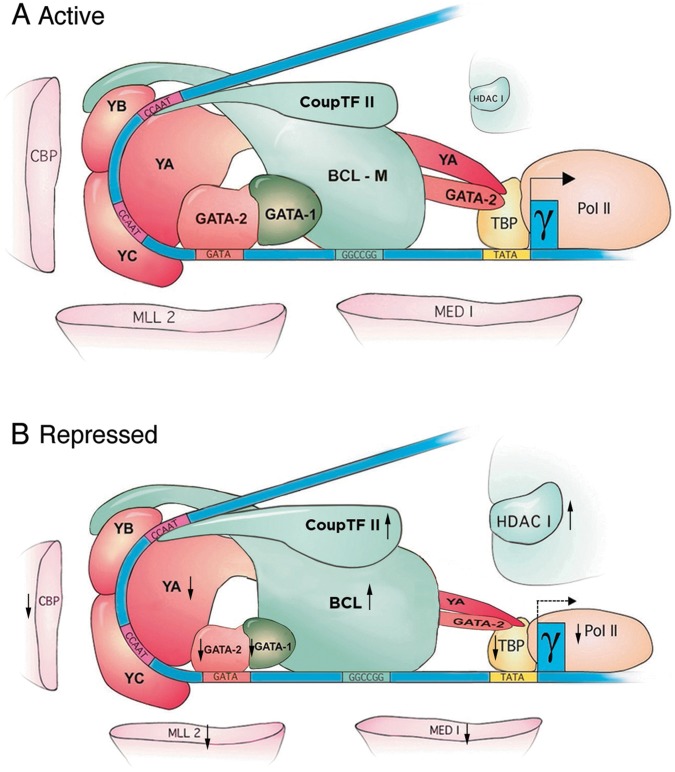
Models of molecular assemblies of the active and repressed proximal γ-globin promoter complexes. A . Active complex. Blue ribbon: Proximal γ-globin promoter DNA containing transcription activator-binding motifs, CCAAT and GATA (red bars) and the repressor-binding motif GGCCGG (green bar); blue rectangle with angled arrow: γ-globin gene and transcriptional direction of γ-globin mRNA. Red colored proteins: transcription activators; pink colored proteins: co-activators. Dark green and green colored proteins: transcription repressors; BCL-M: BCL11A median-isoforms expressed predominantly in human fetal and K562 erythroid cells; light green colored protein: the HDAC1 co-repressor complex. NF-Y bound at each of the tandem CCAAT motifs bends the DNA by ∼70^o^ (3) to form the pocket for assembly of the proximal promoter complex. **B.** Repressed complex. Larger sizes of BCL, COUPTFII and HDAC1 complex and smaller sizes of YA, GATA-2 and -1 and the co-activators represent higher and lower levels of the respective proteins–additionally indicated also by the upward or downward arrows–in the repressed promoter complex as compared to the levels of these proteins in the active complex in **A**; BCL: all sized-isoforms expressed in D14 erythroid cells (Fig. 1C); dotted angled arrow: lower transcription level of γ-globin mRNA from the repressed promoter.

Whether these *in vivo* associations between two target proteins were direct interactions between the two proteins or indirect association through common third protein partners were assessed by *in vitro* co-IP of individual pairs of differently tagged proteins expressed in and isolated from K562 and/or 293 cells. The *in vitro* co-IPs confirmed that most of the protein pairs that associated *in vivo* also associated *in vitro* (Fig. 6B), except that BCL11A, which associated *in vivo* with both NF-YA and -YB (Fig. 6A), associated *in vitro* only with NF–YB (Fig. 6B).

Together, the co-IPs revealed specific protein interactions: (1). Transcription activators NF-Y and GATA-2 interacted with each other to form an apparent activator hub. (2). Repressor BCL11A interacted with COUP-TFII with strong affinity and with GATA-1 (Fig. 6B) to form an apparent repressor hub. (3). The activator and the repressor hubs cross-associated through interactions between activator NF-Y and repressor BCL11A, and activator GATA-2 and repressor GATA-1. (4). The activator hub interacted with co-activators CBP, MLL2 and Mediator 1, and also TBP and pol II in the basal transcription machinery but not with co-repressor HDAC1, while the repressor hub interacted with co-repressor HDAC1 but not the co-activators. Incorporating these and earlier results (Fig. 2,3,4,5,6), the molecular assemblies of the active and the repressed proximal γ-globin promoter complexes were depicted in Fig. 7.

## Discussion

In this study, we showed the pivotal role of NF-Y in assembling the active and the repressed proximal γ-globin promoter complexes (Fig. 7): Transcription activator NF-Y stably bound at the CCAAT motifs with affinity reported to be among the highest for DNA binding proteins [3], [7], [8] recruited and stabilized binding of activator GATA-2 to the neighboring GATA motif to form the activator hub, NF-Y/GATA-2; NF-Y also recruited and stabilized binding to the neighboring GGCCGG motif of repressor BCL11A, which in turn interacted with COUP-TFII bound at its cognate site overlapping the distal CCAAT motif and with GATA-1 recruited by GATA-2 to form the repressor hub, BCL11A/COUP-TFII/GATA-1. The interaction between BCL11A and COUP-TFII in the repressor hub was consistent with earlier reports that BCL11A was originally identified and cloned by its interaction with COUP-TFII and was initially named CTIP1, COUP-TFII Interacting Protein 1 [38] and that it was a transcription repressor with cognate DNA binding motif GGCCGG [11]. The association between BCL11A and GATA-1 has also been previously reported [30]. Due apparently to cross-interaction of NF-Y with BCL11A and GATA-2 with GATA-1, both the activator and the repressor hubs were present in both the active and the repressed γ-globin promoter complexes. However, through their relative levels in the proximal promoter complex and their respective interactions with the co-activators and co-repressor, the activator and the repressor hubs together modulated activation or repression of γ-globin promoter activity during erythroid development.

Our findings shed light on the underlying mechanisms of tissue- and developmental stage-specific transcription of γ-globin gene. In fetal erythroid cells, which expressed at high levels the developmentally regulated, ubiquitous activator NF-Y and erythroid activator GATA-2 (Fig. 1C), NF-Y interacted with GATA-2 to assemble a strong activator hub that recruited co-activators CBP and MLL2 to remodel promoter chromatin, and Mediator 1, TBP and pol II to activate transcription of γ-globin mRNA from the proximal promoter; the fetal erythroid cells also expressed at very low levels repressor BCL11A-M isoforms (Fig. 1C), which assembled apparently a weak repressor hub BCL-M/COUP-TFII/GATA-1 that interacted only weakly with the HDAC repressor complex. The strong activator hub predominated over the weak repressor hub to confer erythroid- and fetal stage-specific transcription of γ-globin mRNA (Fig. 1B). In D14 adult erythorid cells, which expressed lower levels of activators NF-Y and no detectable GATA-2 but much higher levels of all repressor BCL11A isoforms (Fig. 1C), the strong repressor hub predominated over the weakened activator hub to repress transcription of γ-globin mRNA in adult erythroid cells (Fig. 1B). In non-erythroid cells such as brain cells, which expressed a high level of NF-Y and very low to non-detectable levels of GATA-2 (Fig. 1B), but high levels of BCL11A [11] and COUP-TFII [39], the very weak activator hub combined with an apparently strong repressor hub further repressed transcription of γ-globin mRNA in non-erythroid cells (Fig. 1B).

Our finding that BCL11A bound to the GGCCGG motif in the proximal γ-globin promoter was consistent with earlier studies [10], [11]. However, it has been reported that BCL11A does not bind to the γ-globin promoter but binds instead to the locus control region (LCR) far upstream of γ-globin genes and the intergenic region 3′ of Aγ globin gene, thus repressing γ-globin gene transcription indirectly by long-range interactions [30], [40]. The difference in findings may be due in part to sensitivity differences between the techniques of ChIP-chip using microarrays as the quantification tool [40], [41] and the ChIP-qPCR using real-time PCR as the quantification tool employed in this report. In a recent study on global gene expression profiles in human erythroid cells, a side-by side comparison between microarrays and qPCR as quantification tools shows that qPCR is more sensitive in detecting changes in expression levels of genes, including β-globin gene, throughout erythroid development [42]. The human β-globin gene locus contains four GGCCGG binding sites for BCL11A, located respectively near the HS3 site in the LCR, in the proximal promoters of Gγ- and Aγ-globin genes and 3′ of the Aγ-globin gene (GenBank U01317). BCL11A bound to the strong LCR HS3 site at a sufficiently high level detectable by both ChIP-chip and ChIP-qPCR, while it bound to the relatively weak γ-globin promoter site at a lower level detectable only by ChIP-qPCR (Fig. S4). Thus, BCL11A may repress γ-globin gene transcription both by binding to the LCR through an indirect, long-range mechanism and by directly binding to the proximal γ-globin promoter.

The presence of both an activator and a repressor hub in both the active and the repressed proximal γ-globin promoter complex may underlie the propensity of γ-globin promoter to respond to pharmacological compounds that activate the repressed γ-globin gene in adult erythroid cells [1]. The molecular models of the proximal γ-globin promoter complexes (Fig. 7) suggest that these pharmacological compounds may enhance expression of activators, NF-Y and GATA-2, in the activator hub and/or suppress expression of repressors BCL11A, COUP-TFII and GATA-1 in the repressor hub. These possibilities are currently under investigation.

## Supporting Information

Figure S1 The −115 distal CCAAT motif overlapping the COUP-TFII binding site, −73 GATA and −56 GGCCGG did not bind or bound weakly to COUP-TFII, GATA-2 and BCL 11A, respectively, as determined by EMSA (related to Fig. 3). **A.** Percentage contribution of binding by BCL11A to the NF-Y/BCL11A EMSA band. γP wt and γP(CCAAT)m: Wt and mutant CCAAT proximal γ-globin promoter probes. The quantified NF-Y/BCL11A bands were those in Fig. 3A, 3B, 3C, 3D and 3F. The intensities of the control NF-Y/BCL11A band without supershifts by the antibodies were set at 100. **B.** The GGCCGG motif bound BCL11A weakly. Left and middle panels: The short probe spanning −56 GGCCG motif (same sequence as self (GC) competitor in Fig. 3) bound BCL11A very weakly in both K562 and D14 nuclear extracts. Right panel: γP(GC)m (proximal γ-globin promoter with GGCCGG mutated to AAAAAA) bound little BCL11A, since BCL 11A antibodies only slightly decreased the intensity of the NF-Y/BCL band (lanes 8 & 9), indicating requirement of GGCCGG motif to cooperate with CCAAT motif in recruiting and binding of BCL11A. BCL11A Ab-1 and -2: antibodies from Novus NB-100–259 and Abcam Ab19487 respectively. Other designations: same as in Fig. 3. C. COUP-TFII binding to the −115 CCAAT motif overlapping the COUP-TFII site comprised ∼10% of the NF-Y/COUP-TFII EMSA band, as indicated by quantification of the competition bands in Fig. 3D. Competitors dCCAAT and d+p CCAAT spanning both the COUP-TFII and NF-Y sites were 10% more efficient competitors than pCCAAT and E1CCAAT, spanning only the NF-Y site. D. Left panel: The −73 GATA motif by itself did not bind GATA-2/−1. EMSA of short probes spanning −175, a GATA site upstream of the −73 GATA site that bound GATA factors, and the −73 GATA motifs of equal length that did not bind the GATA factors (Compare lanes 1 and 2; for probe sequences, see Methods S1). Right panel: γP(GATA)m, proximal γ-globin promoter with mutated GATA motif, bound GATA-2/−1 much less than the wildtype γP (Compare lanes 3 and 4), indicating cooperativity between GATA and CCAAT motifs in recruiting/binding of GATA-2/−1.
(TIF)

Figure S2 Effect of Mediator 1 and MLL2 knockdown on mRNA levels of γ-globin and select transcription factors in K562 cells. **A.** Knockdown by siRNA targeting MLL2 as well as MLL1 and Menin 1 in the MLL1/2 hCOMPASS-like complex and PTIP in the MLL3/4 hCOMPASS-like complex (22) and Mediator 1 in the Mediator complex [23]. The RNA level of each of the co-factors in K562 cells transfected by the control plasmid producing scrambled siRNA (Si Ctl) was set at 100 to serve as the reference for percentage knockdown of the co-factors by the specific siRNAs. The RNA levels were determined by RT-PCR. **B.** Effects of knockdown of MLL2 and MED1 on γ-globin mRNA level. **C.** Effects of knockdown of MLL2 and MED1 on mRNA levels of select transcription factors and co-factors in the proximal γ-globin promoter complex. The results showed that reduction in transcription of γ-globin gene did not appear to be the secondary effects of MLL2 or MED1 knockdown, which first reduced transcription of activators NF-Y and GATA-2 and/or increased transcription of repressors BCL11A and GATA-1, since NF-Y and GATA-2 levels did not significantly change and the levels of BCL11A and GATA-1actually decreased as a result of MLL2 and MED1 knockdown (Fig. S2C).
(TIF)

Figure S3 Effects of NF-YA knockdown and over-expression of GATA-2 and -1 on molecular assembly of the proximal γ-globin promoter complex in the K562 endogenous genome and in transfected GFP reporter plasmids. **A.** Effects of NF-YA knockdown on assembly of the endogenous proximal γ-globin promoter complex: NF-YA knockdown decreased occupancy of NF-Y, which in turn decreased occupancies of GATA-2 and co-activator MLL2; however, NF-Y knockdown increased occupancy of COUP-TFII, which could competitively bind to its cognate site overlapping the NF-Y binding site at a higher level due to decreased occupancy of NF-Y. On the other hand, occupancy of BCL11A did not correspondingly decrease with a decrease in NF-Y occupancy (Fig. S3A), as anticipated from interaction/association of BCL11A with NF-Y, but increased as a result of the decrease in NF-Y occupancy. This was apparently because BCL11A interacted not only with NF-Y but also strongly with COUP-TFII (11). Thus, an increase in COUP-TFII occupancy increased the recruitment and cccupancy of BCL11A. **B.** Over-expression of GATA-2 and -1 and CCAAT mutation (to abolish NF-Y binding) on assembly of the γ-globin promoter complex in plasmids transiently transfected into K562 cells. 0.13Wt-GFP and 0.13CCAATm-GFP: designations same as in Fig. 2A; Vector: pCRFP1 plasmid containing RFP selectable marker gene; GATA-2 and GATA-1: Expression plasmids containing GATA-1 or -1 cloned into the pCRFP1 vector plasmid. Inset: K562 cells doubly transfected with (GATA-2)-RFP and 0.13 Wt-GFP were sorted by FACS. Sorted cells expressing both RFP and GFP, comprising ∼12% of total cell population, were used for ChIP assays. K562 cells transfected with (GATA-1)-RFP and 0.13 Wt-GFP were similarly sorted by FACS. ChIP results showed that the effects of GATA-2 and -1 over-expression on assembly of the proximal γ-globin promoter complex in transfected plasimds and in the K562 endogenous genome were similar (Compare Fig. S3B with Fig. 4F). In addition, mutation of CCAAT motif (to AACCG, see Fig. 2A) to abolish occupancy of NF-Y greatly diminished occupancies of GATA-2, -1 as well as of BCL11A (Fig. S3A), even though their cognate GATA and GGCCGG binding sites were not mutated. The AACCG mutation eliminated binding of not only NFY but also COUP TFII (Fig. 1A) and would abolish occupancy of COUP-TFII at the proximal γ-globin promoter; in the absence of the protein interaction partners COUP-TFII and NF-Y, BCL11A binding to the GGCCGG motif therefore decreased in the mutant proximal γ- globin promoter.
(TIF)

Figure S4 Relative in vivo binding of BCL11A to the LCR HS3 site and the proximal γ-globin promoter in K562 cells. Values were averages of duplicate pull-downs with the BCL antibody from Novus and Abcam, AB-1 and -2 respectively.
(TIF)

Methods S1 (DOC)
