# Supplementary figures and images for: NF-Y Recruits Both Transcription Activator and Repressor to Modulate Tissue- and Developmental Stage-Specific Expression of Human γ-Globin Gene
Source: PLoS One. 2012 Oct 10;7(10):e47175. doi: 10.1371/journal.pone.0047175 (PMC3468502; doi:10.1371/journal.pone.0047175)

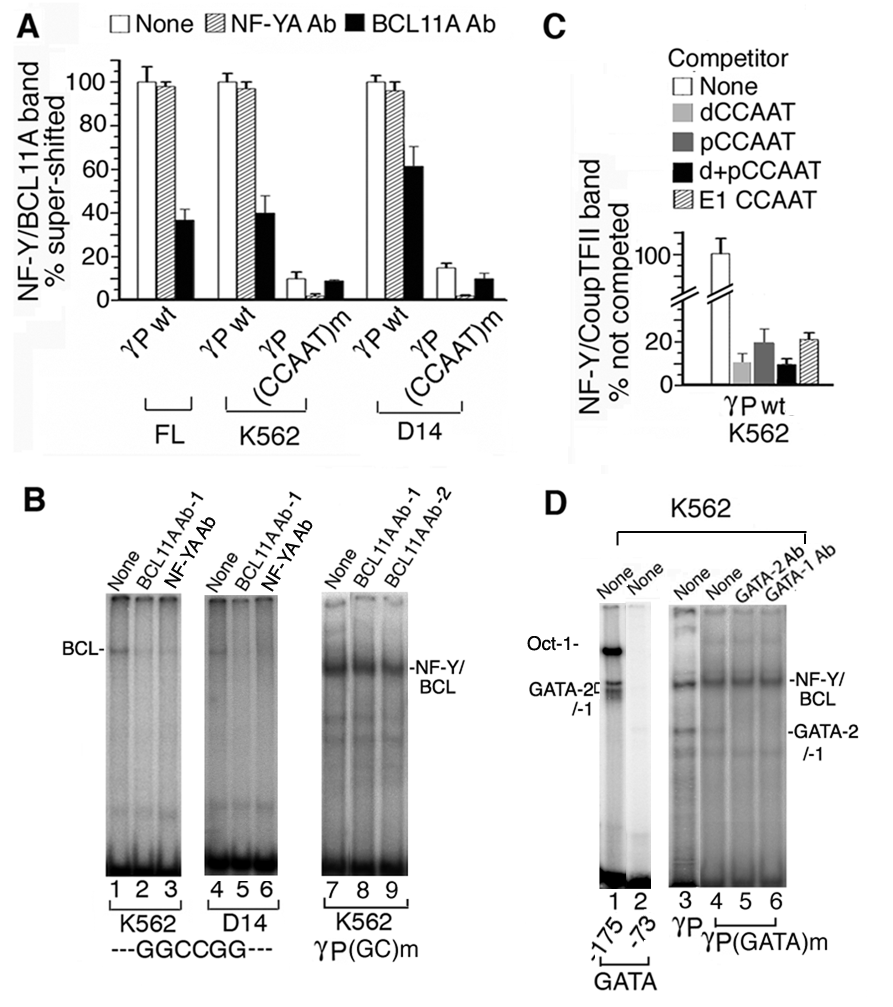

Supplement: Figure S1 — The −115 distal CCAAT motif overlapping the COUP-TFII binding site, −73 GATA and −56 GGCCGG did not bind or bound weakly to COUP-TFII, GATA-2 and BCL 11A, respectively, as determined by EMSA (related to Fig. 3). A. Percentage contribution of binding by BCL11A to the NF-Y/BCL11A EMSA band. γP wt and γP(CCAAT)m: Wt and mutant CCAAT proximal γ-globin promoter probes. The quantified NF-Y/BCL11A bands were those in Fig. 3A, 3B, 3C, 3D and 3F. The intensities of the control NF-Y/BCL11A band without supershifts by the antibodies were set at 100. B. The GGCCGG motif bound BCL11A weakly. Left and middle panels: The short probe spanning −56 GGCCG motif (same sequence as self (GC) competitor in Fig. 3) bound BCL11A very weakly in both K562 and D14 nuclear extracts. Right panel: γP(GC)m (proximal γ-globin promoter with GGCCGG mutated to AAAAAA) bound little BCL11A, since BCL 11A antibodies only slightly decreased the intensity of the NF-Y/BCL band (lanes 8 & 9), indicating requirement of GGCCGG motif to cooperate with CCAAT motif in recruiting and binding of BCL11A. BCL11A Ab-1 and -2: antibodies from Novus NB-100–259 and Abcam Ab19487 respectively. Other designations: same as in Fig. 3. C. COUP-TFII binding to the −115 CCAAT motif overlapping the COUP-TFII site comprised ∼10% of the NF-Y/COUP-TFII EMSA band, as indicated by quantification of the competition bands in Fig. 3D. Competitors dCCAAT and d+p CCAAT spanning both the COUP-TFII and NF-Y sites were 10% more efficient competitors than pCCAAT and E1CCAAT, spanning only the NF-Y site. D. Left panel: The −73 GATA motif by itself did not bind GATA-2/−1. EMSA of short probes spanning −175, a GATA site upstream of the −73 GATA site that bound GATA factors, and the −73 GATA motifs of equal length that did not bind the GATA factors (Compare lanes 1 and 2; for probe sequences, see Methods S1). Right panel: γP(GATA)m, proximal γ-globin promoter with mutated GATA motif, bound GATA-2/−1 much less than the wildtype γP (Compare lanes 3 [file pone.0047175.s001.tif]

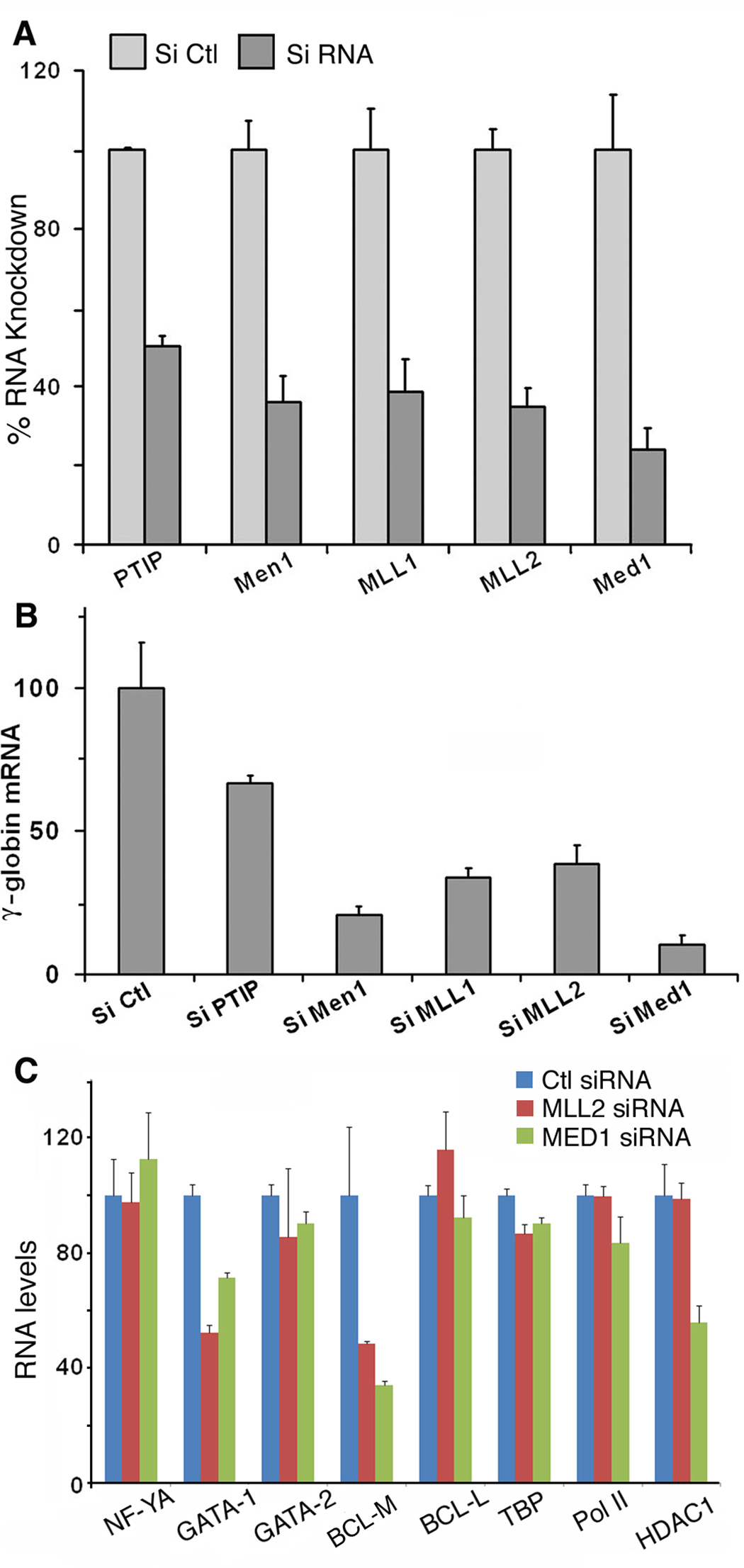

Supplement: Figure S2 — Effect of Mediator 1 and MLL2 knockdown on mRNA levels of γ-globin and select transcription factors in K562 cells. A. Knockdown by siRNA targeting MLL2 as well as MLL1 and Menin 1 in the MLL1/2 hCOMPASS-like complex and PTIP in the MLL3/4 hCOMPASS-like complex (22) and Mediator 1 in the Mediator complex [23]. The RNA level of each of the co-factors in K562 cells transfected by the control plasmid producing scrambled siRNA (Si Ctl) was set at 100 to serve as the reference for percentage knockdown of the co-factors by the specific siRNAs. The RNA levels were determined by RT-PCR. B. Effects of knockdown of MLL2 and MED1 on γ-globin mRNA level. C. Effects of knockdown of MLL2 and MED1 on mRNA levels of select transcription factors and co-factors in the proximal γ-globin promoter complex. The results showed that reduction in transcription of γ-globin gene did not appear to be the secondary effects of MLL2 or MED1 knockdown, which first reduced transcription of activators NF-Y and GATA-2 and/or increased transcription of repressors BCL11A and GATA-1, since NF-Y and GATA-2 levels did not significantly change and the levels of BCL11A and GATA-1actually decreased as a result of MLL2 and MED1 knockdown (Fig. S2C). (TIF) [file pone.0047175.s002.tif]

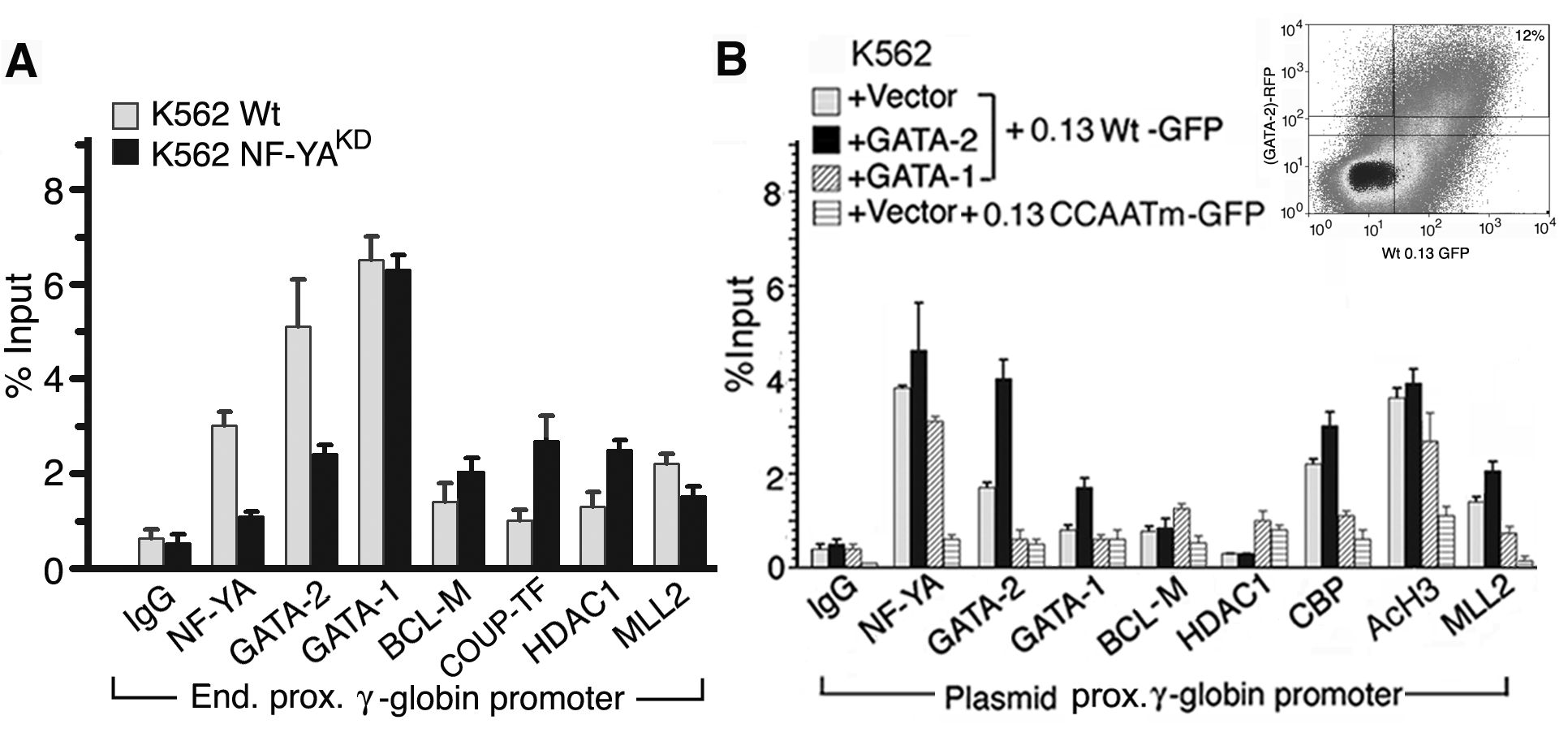

Supplement: Figure S3 — Effects of NF-YA knockdown and over-expression of GATA-2 and -1 on molecular assembly of the proximal γ-globin promoter complex in the K562 endogenous genome and in transfected GFP reporter plasmids. A. Effects of NF-YA knockdown on assembly of the endogenous proximal γ-globin promoter complex: NF-YA knockdown decreased occupancy of NF-Y, which in turn decreased occupancies of GATA-2 and co-activator MLL2; however, NF-Y knockdown increased occupancy of COUP-TFII, which could competitively bind to its cognate site overlapping the NF-Y binding site at a higher level due to decreased occupancy of NF-Y. On the other hand, occupancy of BCL11A did not correspondingly decrease with a decrease in NF-Y occupancy (Fig. S3A), as anticipated from interaction/association of BCL11A with NF-Y, but increased as a result of the decrease in NF-Y occupancy. This was apparently because BCL11A interacted not only with NF-Y but also strongly with COUP-TFII (11). Thus, an increase in COUP-TFII occupancy increased the recruitment and cccupancy of BCL11A. B. Over-expression of GATA-2 and -1 and CCAAT mutation (to abolish NF-Y binding) on assembly of the γ-globin promoter complex in plasmids transiently transfected into K562 cells. 0.13Wt-GFP and 0.13CCAATm-GFP: designations same as in Fig. 2A; Vector: pCRFP1 plasmid containing RFP selectable marker gene; GATA-2 and GATA-1: Expression plasmids containing GATA-1 or -1 cloned into the pCRFP1 vector plasmid. Inset: K562 cells doubly transfected with (GATA-2)-RFP and 0.13 Wt-GFP were sorted by FACS. Sorted cells expressing both RFP and GFP, comprising ∼12% of total cell population, were used for ChIP assays. K562 cells transfected with (GATA-1)-RFP and 0.13 Wt-GFP were similarly sorted by FACS. ChIP results showed that the effects of GATA-2 and -1 over-expression on assembly of the proximal γ-globin promoter complex in transfected plasimds and in the K562 endogenous genome were similar (Compare Fig. S3B with Fig. 4F). In addition, mutation of CC [file pone.0047175.s003.tif]

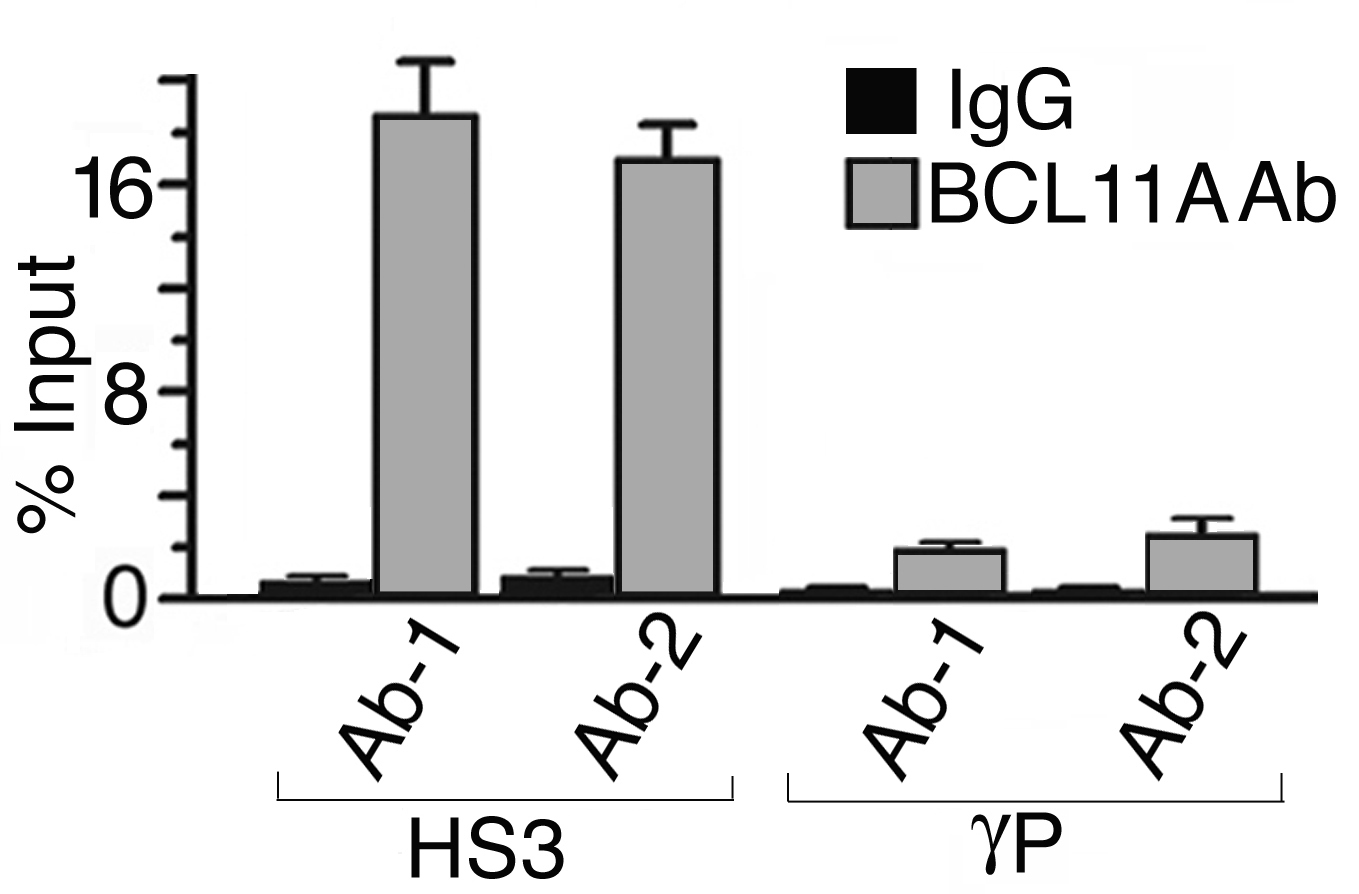

Supplement: Figure S4 — Relative in vivo binding of BCL11A to the LCR HS3 site and the proximal γ-globin promoter in K562 cells. Values were averages of duplicate pull-downs with the BCL antibody from Novus and Abcam, AB-1 and -2 respectively. (TIF) [file pone.0047175.s004.tif]
