# Supplementary material for: NF-Y Recruits Both Transcription Activator and Repressor to Modulate Tissue- and Developmental Stage-Specific Expression of Human γ-Globin Gene
Source: PLoS One. 2012 Oct 10;7(10):e47175. doi: 10.1371/journal.pone.0047175 (PMC3468502; doi:10.1371/journal.pone.0047175)
Supplement: Methods S1 — (DOC) [file pone.0047175.s005.doc]

**Supplemental Methods**

**Plasmids:**

The 1.3 and 0.13 γp-GFP plasmids for transfection/transduction into K562 and D14 erythroid cells (Fig. 2B and 2C): The plasmids for transfection into K562 cells were constructed in pEGFP-C vector (Clontech). The Wt and mutant 1.3 kb and 0.13 kb γ-globin promoter were amplified by PCR from genomic DNA and inserted into the Ase1 and Nhe1 sites 5’ of GFP gene. Base mutations in the 1.3 kb long and the 0.13 proximal promoters were introduced by PCR primers containing the respective mutations. The 0.13 γp-GFP lentiviral plasmid for transduction of human primary adult erythroid cells (Fig. 2C) was constructed in lentiviral vector pCDF1-MCS2-EF1-copGFP (System Biosciences).

Expression plasmids for myc-GATA-1 and -2 (Fig. 3) were constructed in pEGFP-C vector in which the cDNA for GATA-1 (NM_002049) or -2 (NM_032638) replaced GFP gene; the myc-tag was expressed at the N-terminus of the GATA proteins.

Lentiviral plasmids for over-expression and siRNA knockdown of transcription factors and co-factors (Fig. 4, S2 and S3): The cDNAs for over-expressing NF-YAL and –YAS isoforms, GATA-1 or -2 were cloned into the MCS site of lentiviral vector pCDF1-MCS2-EF1-copGFP (Fig. 4) or into the MCS site of pCRFP1 (Fig. S3), which contained the same vector backbone as pCDF1-MCS2-EF1-copGFP. The lentiviral plasmids for siRNA knockdown of NF-YA, GATA-1 and -2 (Fig. 4), MLL2, MLL1, Menin 1, PTIP and Mediator 1 (Fig. S2) and the control scrambled RNA UAUCUCUUCA-UAGCCUUA were constructed in pLentilox 3.7 vector. For over-expression of BCL11A isoforms (Fig. 4), the cDNAs for BCL11A-XL, -L, -M and -S isoforms were synthesized from D14 total cellular RNAs and cloned as a GFP-fusion gene into pEGFP-C plasmid (Clontech).

Plasmids for over-expression of FLAG-NF-Y, HA-GATA-2, His-GATA-1 and myc-BCL11A-XL (Fig. 6A): The cDNA for NF-Y, GATA-2 or-1, or BCL11A-XL was cloned into pEGFP-C plasmid (Clontech), replacing GFP gene; the tags were expressed at the N-termini of the proteins.

Plasmids for protein expression for in vitro co-IP (Fig. 6B): The cDNA plasmid for human COUPTFII, pcDNA5-hCOUP-TFII, was a gift from Dr. S. Tsai, for N-terminal MLL2, pMSCV-fMLL2-pL, was a gift from Dr. R. Slany. The cDNA for MLL2 (C-terminal), CBP, HDAC1, MED1 and TBP were from Addgene, # 11017, 16701, 13820, 17433 and BC016476 respectively, which were cloned into pEGFP-C3 (Clontech) as a GFP fusion protein.

**Packaging of recombinant lentiviruses and transduction of erythroid cells:** Recombinant lentiviruses were packaged as described (Nature Genetics 33, 401-406, 2003). Briefly, the pCRFP1 plasmid over-expressing NF-YA, GATA-2, -1 and pLL3.7 plasmid expressing GATA-1 siRNA was co-transfected with helper plasmids into 293T cells; the viral supernatant was collected after 36-48hrs; the recombinant lentiviruses in the supernatant were titrated and concentrated by using **Lenti-X qRT-PCR Titration Kits (**Clontech, **Cat#** 631235**) and** Lenti-X Concentrator (Clontech, Cat# 631231). The concentrated lentiviral particles were mixed with K562 or Day 3 adult human erythroid progenitor cells at a multiplicity of infection (MOI) of ~50 in the presence of 8ug/ml Polybrene and incubated overnight at 37^o^C. The viral supernatant was removed after 12-16hrs and the K562 were cultured for 72 hours and the primary adult human erythroid cells for 4-10 additional days, when they were collected for FACS sorting with GFP fluorescence.

**EMSA and Immuno-depletion (ID)-EMSA** (Fig. 3)**:** EMSAs were carried out as described (25). For ID-EMSA the NF-YA, GATA-1 or GATA-2 proteins were immuno-depleted from K562 nuclear extract (NE) with NF-YA, GATA-1 or GATA-2 antibodies (SC-10779x, SC-1233x, SC-1235x) or with normal rabbit IgG (Cat: NI01, Calbiochem), as described (Nucl Acids Res, 23, 3345-3346, 1995). In brief, 400ug K562 N.E. was incubated with 2-5ug antibodies or normal rabbit IgG overnight while rotating at 4^0^C. Protein A/G beads (200ul) were added to the complex and rotated for 3hrs. The protein/antibody-A/G beads complex was spun down and removed. Another aliquot of 200ul Protein A/G beads was added to the supernatant to deplete the remaining traces of antibodies. The immuno-depletion was confirmed by Western blot using corresponding NF-YA, GATA-1 or GATA-2 antibodies; NF-YA, GATA-1 or GATA-2 depleted K562 N.E. (4ug) were used for the ID-EMSAs (Fig. 3H).

**In vivo co-IP:** In vivo co-IP (Fig. 6A) was carried out using an Abcam protocol. Briefly, K562 cells were transfected with plasmids over-expressing FLAG-NF-YA, HA-GATA-1, His-GATA-2 or Myc-BCL11A-XL. After 48-72 hours, cells were collected and lyzed. Proteins associated with the tagged NF-YA, GATA-2 or -1 or BCL11-XL were immuno-precipitated by the antibodies against the respective tags using 0.5-2ug antibodies per 500ug proteins in the K562 cell lysates. The immuno-precipitated proteins were identified by Western blots.

**In vitro co-IP:** The proteins of interest were expressed from expression plasmids transfected into K562 and/or HEK 293 cells. Forty-eight hours after transfection, cells were lyzed with NP40 cell lysis buffer and the supernatants were collected. The supernatant lyzate was adjusted to 800mM NaCl and the lysate incubate at 4^o^C for 3hrs. Normal rabbit IgG and protein A/G agarose beads were added to pre-clean the lysate. After this, the appropriate antibody against the protein was added to the lysate. The lyzate was subsequently divided into two aliquots. Protein A or G agarose beads (Millipore, Cat# 16-156, 16-266,) was added to one aliquot and protein A or G magnet Dynabeads (Invitrogen, Cat: 100.01D and 100.03D) added to the second aliquot. Both aliquots were rotated at 4^o^C for 3-4hrs. The protein-agarose beads and protein-Dynabeads were washed 2x with 10 mM Tris pH 8 containing 800mM NaCl, and resuspended in 10 mM Tris pH 8, 150mM NaCl. To study the in vitro interaction between specific pairs of proteins, for example protein-X and protein-Y, aliquot of protein-X attached to the A/G agarose beads was mixed with aliquot of protein-Y attached to the magnet Dynabeads, serving as bait. The protein mixture was separated on the magnet after incubation overnight at 4^o^C. The mixture of protein-X-agarose and protein-Y-Dynabeads attached to the magnet was washed with NP40 cell lysis buffer 3x before the protein mixture was released from the magnet in SDS buffer (50mM Tris-HCl, pH 6.8, 4% SDS, 20% glycerol, 2% 2-mercaptoethanol, and bromophenol blue)**.** The bait protein-Y and the prey protein-X were separately detected by their respective specific antibodies in Western blots.

**Antibodies:** If not otherwise specified, the antibodies were from Santa Cruiz Biotechnology.

EMSA (Fig. 3): Anti-NFYA (SC10779x), anti-GATA1 (SC1233x), anti-GATA2 (SC1235x), anti-BCL11A (NB100-258, Novus), anti-Sp1 (07-645, Millipore), anti-CP2c (ab42973, Abcam).

ChIP assays: The EMSA antibodies and anti-CBP (SC369), anti-COUPTFII (ab64849, abcam), anti-H3K4Ac (07-539, Millipore), anti-H3K4Me3 (ab8580, Abcam), anti-HDAC1 (SC7872), anti-MED1 (SC8998), anti-MLL2 (A300-113A, Bethyl), anti-NFE4 (rabbit IgG, a kind gift from Dr. S. Jane), anti-PolII (MMS-126R, Covance), anti-Sirt1 (SC15404), anti-TBP (SC273).

Western blots and co-IP: The antibodies as described above, and anti-FLAG (F3165, Sigma), anti-GFP (G1544, Sigma), anti-HA (H9658, Sigma), anti-His (H1029, Sigma), anti-MYC (9E10, Santa cruz), anti-β actin (A5316, Sigma), anti-HDAC2 (SC7899), anti-Mi2 (SC11378), anti-GATA1 (SC13053), anti-GATA2 (SC9008).

**EMSA probes and competitors:**

Probes

Wt proximal γ-globin probe spanning base positions 34350-34438 in GenBank file U01317 and the mutant probes were annealed from single oligonucleotides of 70 bases. The annealed, double stranded probes contained 5’ and 3’ single stranded overhangs of 20 bases, which were filled in with dNTPs containing ^32^P-dATP using Klenow enzyme as described (Yu et al., 2005).

γP(GC)m: Base mutations were in bold and underlined.

GCCTTGCCTTGACCAATAGCCTTGACAAGGCAAACTTGACCAATAGTCTTAGAGTATCCAGTGAGGCCAGGGG**AAAA**CGGCTGGCTAGGGATGA

γP(GATA)m: GCCTTGCCTTGACCAATAGCCTTGACAAGGCAAACTTGACCAATAGTCTTAGAGTA**C**CCAGTGAGGCCAGGG

Competitors

(GATA)7: ATGAAAGAATAGATAGATAGATAGACAGATAGATGATAGATAGAATAAATG

(in HS5 site of the human β-globin LCR, Ramchandran et al., 1999)

-175 GATA: TCAATGCAAATATCTGTCTGAAACG (in γ-globin promoter)

-73 GATA: TCTTAGAGTATCCAGTGAGGCCA

Self (GC): [AGTGAGGCCAGGGGCCGGCGGCTGGCT](https://www.idtdna.com/OrderStatus/SpecSheet.aspx?OrderNum=070000000BE82F9F9E15AD2A7CAD989C877E64C9&MfgID=08000000CCFA438379F3846439613FB386A0C521&MfgLocID=1&ProdID=1213&position=0)AGGGATGAA

dCCAAT: CCTTGCCTTGACCAATAGCCTTGACAAG (distal CCAAT motif)

pCCAAT: AGGCAAACTTGACCAATAGTCTTAGAG (proximal CCAAT motif)

E1(CCAAT): GTAAATACACCAATCGGCAGTCTGAAG (Yu et al., 2005)

**PCR, RT-PCR and ChIP primers:**

Synthesis of cDNAs

NF-YAL (NM_002505): F, gctagcCATGGAGCAGTATACAGCAAAC

R, tcAGGACACTCGGATGATCTGTGTCAT;

NF-YAS (BC039244): Same as for NF-YAL

BCL-XL(NM_022893) F, TAGATCTCGAGCCGCCACCATGTCTCGCCGCAAGCAAG; R, atggatccCTATTCAGTTTTTATATCATTATTC;

BCL-L (NM_018014) F, Same as that for BCL11A-XL; R, cctttaaAACTTAAGGGCTCTC;

BCL-M F & R, Same as those for BCL-L

BCL-S (NM_138559) F, same as that for BCL11A-XL;

R, tcagatcTTCTCAGAACTTAAGGGCTC;

(lower case letters: extra bases including restriction enzyme sites added as cloning sites)

RT-PCR:

α-globin: F, 51-74; R, 466-443, NM_000558

β-globin: F, 62611-62629, R, 63720-63695, U01317

ε-globin: F, 19957-19575, R, 21045-21021, U01317

γ-globin: F, 34947-34565, R, 36033-36009, U01317

NF-YA: F, 797-819, R, 1111-1091, NM_002505 (for both YAL and YAS isoforms)

GATA-1: F, 297-319, R, 755-733, NM_002049

GATA-2: F, 775-797, R, 1396-1374, NM_032638

BCL11A: F, 224-247, R, 456-436, NM_022893 (for all isoforms)

18s rRNA: F, 1464-1483, R, 1715-1696, NR_003286

Mediator1(TRAP220): F, CCAGCATGGGAGTTCTAAAGGAA

R, TCCATCATGCTTACTGATGATAA

Menin1: F, CCGTGGCCGACCTGTCTATCATC

R, CCCAAACACTACCCAGGCATGAT

MLL1: F, CTGCACCAGCAACTATCACTTCA

R, CGAGCATCTGTGGTGCTCCAGTA

MLL2 F, CCAGACGTGCCACCAGCCTGGAG

R, CGAGAGAAGCAGTTGGGCTCACA

PTIP F, TTGTGACTCCTGACTGGGTTCTG

R, GCTGCAGCTAACTGTGGGACTTC

ChIP:

Endog. proximal γ-globin promoter: F, 34352-34380, R, 34588-34566, U01317

**siRNAs target sequences:**

NF-YA: GCAGGCAATGTGGTCAAT

GATA2: CTTCCCACCCACGCCACC

GATA1: TGGCGGAGGGACAGGACA

MLL2: CATGGATGACTTTGATGT

MLL1: TTCCTGAGAATGGATTTG

Men1: GGGGTGTCTCCAGCCGTG

PTIP: CCTGATCTCATTGGACTT

Mediator1: GAGCACAAATTCTTCCAG

Scrambled siRNA: TATCTCTTCATAGCCTTA
